# Pioneering Insights into the Reaction Kinetics of Metastable Intermolecular Composites Based on Metal Fluorides: Virtually non‐existent condensed Phase Combustion Products and Ultra‐Efficient Reactivity

**DOI:** 10.1002/advs.202415073

**Published:** 2025-02-18

**Authors:** Xuwen Liu, Jingwei Li, Shenghua Feng, Yongsheng Jia, Maocong Hu, Yingkang Yao, Jinshan Sun, Quanmin Xie, Hongqian Sang

**Affiliations:** ^1^ State Key Laboratory of Precision Blasting Jianghan University Wuhan 430113 China; ^2^ Hubei Key Laboratory of Blasting Engineering Jianghan University Wuhan 430056 China; ^3^ School of Chemical and Blasting Engineering Anhui University of Science and Technology Huainan 232001 China; ^4^ School of Optoelectronic Materials and Technology Jianghan University Wuhan 430056 China

**Keywords:** combustion mechanism, metal fluorides, metastable intermolecular composites, nanostructured energetic materials, pre‐ignition reactions

## Abstract

As a typical representative of Metastable intermolecular composites (MICs), the energy release of nano‐thermites relying on aluminum‐oxygen reaction is limited by the formation of high boiling point condensed phase products. Low pressure output performance constitutes another pivotal factor influencing their efficacy. In this work, metal fluorides BiF_3_ at different scales were incorporated into nano‐thermites as oxidants, thereby facilitating the tunability of the released energy. The boiling points of all resultant reaction products fall below the combustion temperature, theoretically abolishing the agglomeration of condensed‐phase products, thus preventing the entrapment of active metals. Additionally, it facilitates the smooth conduction of heat flux, thereby averting losses in biphasic flow dynamics. The n‐Al/n‐BiF_3_ system exhibits a significant amplification in reactive kinetic properties in stark contrast to the n‐Al/n‐Bi_2_O_3_ system. The reduction in ignition threshold is ascribed to a novel reaction kinetics mechanism within the n‐Al/BiF_3_ system. The highly electronegative fluorine within BiF_3_ corrodes the Al_2_O_3_ shell, inducing a “pre‐ignition” reaction. The application of Density Functional Theory (DFT) evaluations has further corroborated the n‐Al/n‐BiF_3_ system's preeminence in electron transfer capacity between the oxidizing agent and fuel, thereby furnishing an molecular‐electronic basis for its potent reactive kinetic properties.

## Graphical abstract

Novel nano‐thermite designed based on bismuth's low boiling point and the Bi─F bond's low dissociation energy, features enhanced reactivity, minimal condensed phase combustion residues, and self‐pressurization. Its distinctive reaction dynamics facilitate a pre‐ignition stage, resulting in a reduced ignition threshold.

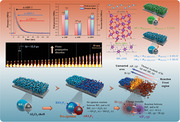

## 1 Introduction

Nano‐thermites, characterized as composite materials consisting of nanoscale metallic fuels and oxidizers, are recognized as a typical class within the domain of metastable intermolecular composites (MICs).^[^ ^1^, ^2^, ^3^, ^4^, ^5^ ^]^ These composites are notable for their significant energy density, minimal diffusion scale, and extensive reactive interface, which endow them with superior properties such as heightened reactivity, low ignition threshold, and swift reaction kinetics.^[^ ^2^, ^3^, ^4^ ^]^ With appropriate energetic input, nano‐thermites are capable of initiating rapid redox reactions, leading to the release of considerable amounts of energy.^[^ ^5^, ^6^ ^]^ This capability has elicited substantial interest among researchers in various fields, including the development of propellants, explosives, ignition mechanisms, micro/nanosatellite attitude control systems, and methods for minimally invasive sterilization.^[^ ^4^, ^5^, ^6^, ^7^, ^8^ ^]^

However, the utilization of nano‐thermites faces several challenges. During the oxidation process of aluminum, a high‐boiling‐point product, Al_2_O_3_ (2980 °C), forms a dense layer on the aluminum surface.^[^ ^9^, ^10^, ^11^ ^]^ This layer acts as a barrier to further reaction, facilitates the sintering of aluminum, and reduces the kinetic advantages inherent in the system.^[^ ^10^, ^11^, ^12^ ^]^ Additionally, the formation of a dense oxide shell on aluminum nanoparticles (Al NPs) negatively affects the ignition capacity of the system and extends the ignition delay.^[^ ^9^, ^10^, ^11^ ^]^ Furthermore, the reaction products of traditional thermites predominantly consist of high‐boiling‐point oxides and reductive metallic elements, such as Cu (2567 °C), Fe (2750 °C), and Mo (5560 °C), without significant generation of gases.^[^ ^13^, ^14^ ^]^ This leads to nano‐thermite systems releasing energy primarily in the form of heat, rather than facilitating work through the generation of pressure, thereby posing challenges in achieving desired outcomes like high pressure or shock waves.^[^ ^13^, ^14^ ^]^

The integration of fluorides into nano‐thermite is identified as a viable approach to amplify their performance.^[^ ^15^, ^16^ ^]^ The pronounced electronegativity of fluorine imparts substantial enhancements in its interaction with aluminum. Specifically, through pre‐ignition reactions (PIR), fluorine engages with the Al_2_O_3_ shell prior to the attainment of the ignition threshold, thus unveiling the highly reactive aluminum nucleus and subsequently augmenting the ignition efficacy of the system.^[^ ^11^, ^17^ ^]^ Moreover, fluorides contribute to the generation of a lower‐boiling‐point product, AlF_3_ (1537 °C), which serves to bolster thermal convection within the reactive milieu, effectively addressing the aluminum sintering dilemma.^[^ ^16^, ^17^, ^18^ ^]^ Additionally, the bond energy associated with the Al─F bond (675 kJ mol^−1^) surpasses that of the Al─O bond (502 kJ mol^−1^), facilitating a greater energy release per mole of aluminum upon the formation of fluoride products (AlF_3_, 1510 kJ mol^−1^) compared to oxide products (Al_2_O_3_, 834 kJ mol^−1^).^[^ ^15^ ^]^

In the domain of nano‐thermite formulations, the oxidizers frequently encompass fluorocarbon polymers, such as polyvinylidene fluoride, polytetrafluoroethylene, and fluorinated alkyl substances (FAS).^[^ ^15^ ^]^ Their assimilation into nano‐thermite constructs is shown to elevate ignition and pressurization capacities.^[^ ^19^ ^]^ However, the robust bond energies characteristic of C─F bonds, coupled with the prevalence of C─C bonds within these polymers, may attenuate the thermodynamic advantages during the reaction process.^[^ ^15^ ^]^ Furthermore, it has been observed that the inclusion of fluorocarbon polymers may also diminish the kinetic benefits traditionally associated with nano‐thermite systems.^[^ ^16^, ^20^ ^]^

In the landscape of fluorine‐based oxidizers, inorganic fluorides stand out as a burgeoning category, distinct from the well‐examined fluoropolymers, and have garnered the interest of the research community. Dreizin and colleagues have embarked on investigations into the amalgamation of various transition and post‐transition metal fluorides with micron‐sized metals such as aluminum and boron. This exploration was conducted via mechanical ball milling, aiming to assess their utility as enhancers in propellant formulations.^[^ ^21^, ^22^, ^23^ ^]^ Moreover, nano‐thermite constructs employing inorganic metal fluorides, notably Al/CuF_2_,^[^ ^24^ ^]^ Al/FeF_x_,^[^ ^25^, ^26^ ^]^ Al/BiOF,^[^ ^18^ ^]^ and Al/Co(OH)F,^[^ ^27^ ^]^ have demonstrated remarkable efficacy in recent studies over the past two years.

Consequently, this study is predicated on the development of a nano‐thermite system characterized by low agglomerate phase products, high pressurization attributes, and a low ignition threshold, leveraging the properties of Bi's low boiling point (1564 °C) and the low bond energy of Bi─F (259 kJ mol^−1^).^[^ ^15^ ^]^ Bismuth trifluoride (BiF_3_) was selected as the fluorine‐containing oxidizer within the system. Differentiated synthesis processes were employed to fabricate BiF_3_ at both microns (μ‐BiF_3_) and nanoscales (n‐BiF_3_), upon which the nano‐thermite system was constructed. Investigations were conducted on its ignition and flame propagation performance under open conditions, energy release characteristics under confined conditions, combustion temperature, ignition temperature, and pressure output capability. Comparisons were made with the corresponding oxide system (Al/Bi_2_O_3_), and its reaction mechanism was analyzed through thermal analysis and a series of product characterization techniques. Finally, the electron transfer capabilities of Al/BiF_3_ and Al/Bi_2_O_3_ systems were theoretically analyzed using DFT calculations.

## 2 Results and Discussions

### 2.1 Morphology

**Figure 1:**
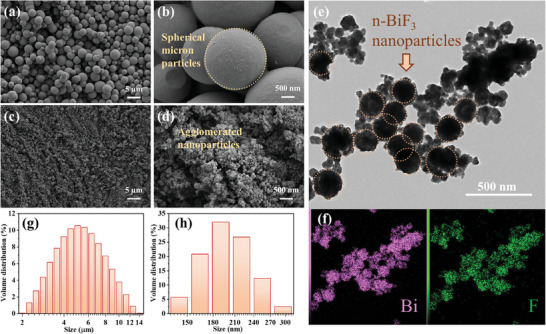
Microstructural characterization of bismuth trifluoride. SEM images of synthesized a,b) μ‐BiF_3_ and c,d) n‐BiF_3_; e,f) HRTEM and corresponding element distribution images of n‐BiF_3_; μ Laser particle size distribution of g) μ‐BiF_3_ and h) n‐BiF_3_.

To investigate the morphological characteristics of the synthesized BiF_3_, SEM was employed to characterize the samples. **Figure** **1**a,b displays the SEM images of µ‐BiF_3_, from which it can be observed that µ‐BiF_3_ exhibits micrometer‐scale spherical particle morphology with good sphericity, and the uniformity of particle size shows no significant variation. Conversely, Figure 1c,d presents the SEM images of n‐BiF_3_, revealing that n‐BiF_3_ consists of agglomerated nanoparticles at the microscale. To characterize the microstructure of n‐BiF_3_ in greater detail, HRTEM technology was utilized. Figure 1e,f showcases the HRTEM experimental results of n‐BiF_3_, clearly depicting the micro‐morphological characteristics of the agglomerated spherical particles of n‐BiF_3_. The corresponding elemental mapping results also indicate the uniform distribution of Bi and F elements on the particles. To further quantitatively evaluate the particle size distribution of the two synthesized types of BiF_3_, laser particle size analysis was conducted, with the results shown in Figure 1g,h. According to the particle size distribution curves, both µ‐BiF_3_ and n‐BiF_3_ exhibit a normal distribution, with average particle sizes of 5.49 µm and 201 nm, respectively.

**Figure 2:**
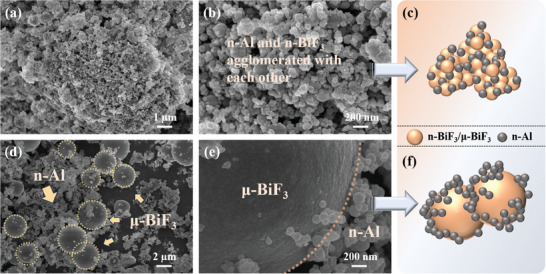
Microstructural characteristics of n‐ABF and µ‐ABF systems. a,b) SEM images and c) schematic structural diagram of n‐ABF; d,e) SEM images and f) schematic structural diagram of µ‐ABF.

**Figure** **2**a,b displays the SEM images of n‐ABF, revealing a well‐integrated mixture of Al NPs and n‐BiF_3_ particles. The formation of this homogeneous state is facilitated by the small size of the n‐BiF_3_ particles, which promotes enhanced interfacial coupling with Al NPs. Utilizing ultrasonic dispersion, Al NPs are embedded within the interstices of the n‐BiF_3_ particles, thereby improving interfacial contact, as illustrated in Figure 2c. Figure 2d,e depicts the SEM images of the μ‐ABF system, which demonstrate distinct microstructural characteristics. Due to the substantially larger size of the μ‐BiF_3_ particles relative to Al NPs, the nanoscale Al NPs adhere to the surfaces of the micrometer‐scale μ‐BiF_3_ particles, as shown in Figure 2f.

### 2.2 Phase Characterizations

**Figure 3:**
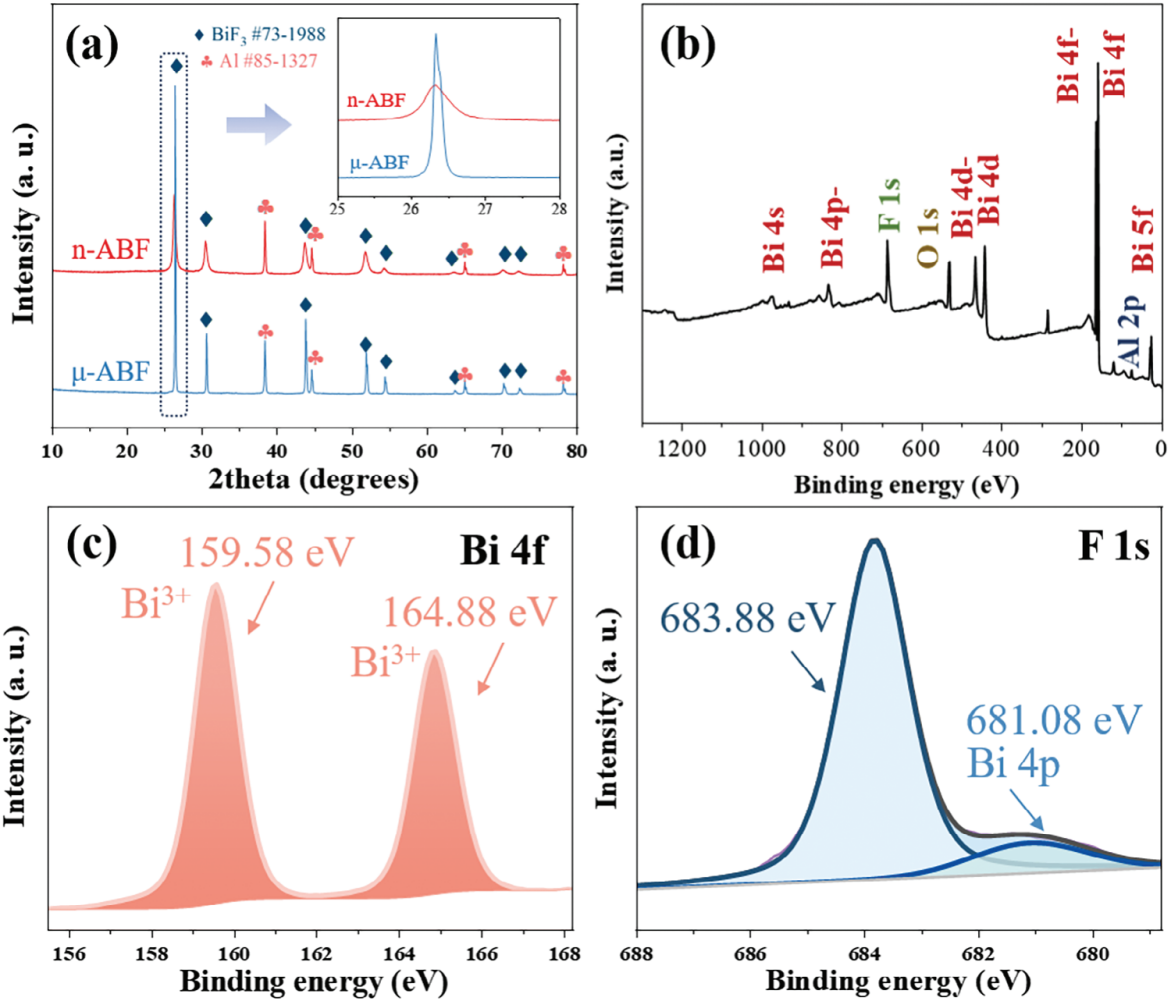
Phase and surface valence state characteristics of thermite system. a) XRD spectrum, b) XPS total spectrum, c) Bi 4f high‐resolution spectrum, and d) F 1s high‐resolution spectrum of n‐Al/BiF_3_.

The crystal phases and surface chemical valence states of the n‐Al/BiF_3_ system were analyzed through XRD and XPS. **Figure** **3**a presents the XRD spectra of the n‐ABF and μ‐ABF system, where the diffraction peaks observed at 38.47°, 44.72°, 65.10°, and 78.23° correspond to the (200), (220), (311), and (222) planes of Al (JCPDS No. 85–1327), respectively. Additionally, the diffraction peaks at 26.32°, 30.48°, 43.64°, 51.68°, 54.17°, 63.43°, 69.90°, and 71.99° correspond to the (111), (200), (220), (311), (222), (400), (331), and (420) planes of BiF_3_ (JCPDS No. 73–1988).^[^ ^28^ ^]^ In addition, from the XRD spectrum (partial view in Figure 3a), it can be observed that n‐ABF has a peak broadening characteristic relative to μ‐ABF, indicating that n‐ABF has smaller grains.

Figure 3b–d displays the XPS spectra for the n‐Al/BiF_3_. The survey spectrum in Figure 3b reveals that the sample comprises elements Bi, Al, O, and F. The high‐resolution Bi 4f spectrum shown in Figure 3c features two prominent spin–orbit splitting peaks located at binding energies of 159.58 and 164.88 eV, corresponding to the Bi 4f 7/2 and Bi 4f 5/2 signals, respectively. This indicates the trivalent oxidation state of Bi.^[^ ^29^ ^]^ Figure 3d presents the high‐resolution F 1s spectrum, where the peak at a binding energy of 683.88 eV corresponds to the monovalent oxidation state of F. The peak at a binding energy of 681.08 eV is attributed to Bi 4p.

### 2.3 Unrestrained Combustion Behavior

**Figure 4:**
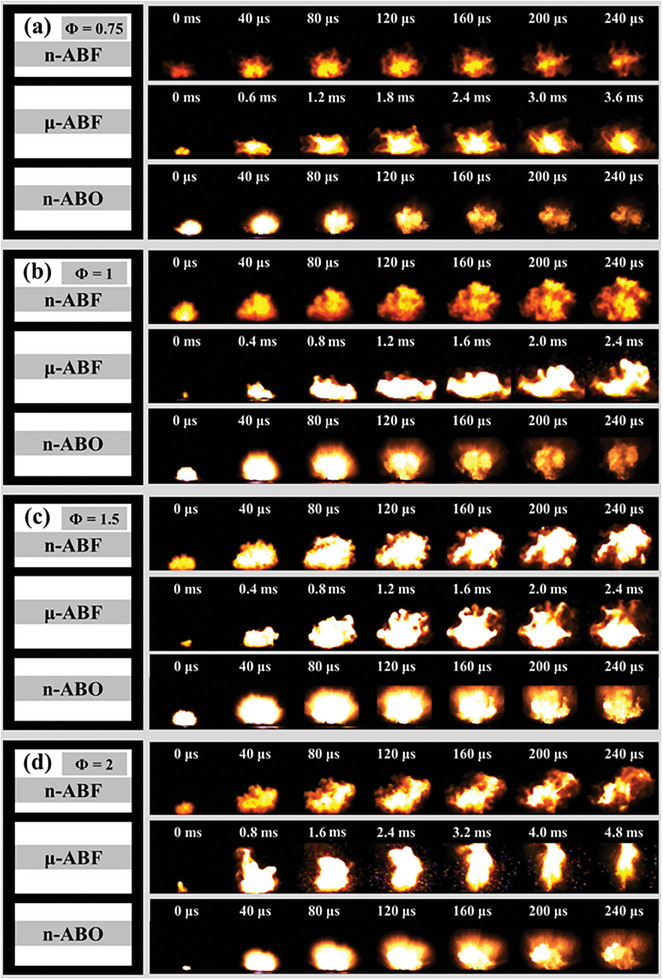
Combustion behavior of n‐Al/BiF_3_ and n‐Al/Bi_2_O_3_ systems under different *Φ* conditions. a) *Φ* = 0.75; b) *Φ* = 1; c) *Φ* = 1.5; d) *Φ* = 2.

**Figure 5:**
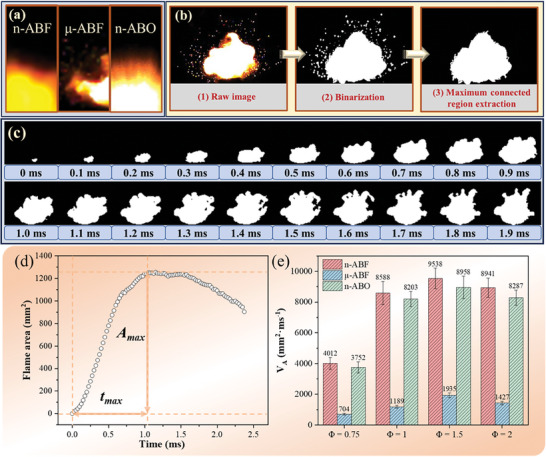
Flame growth characteristics of each nano‐thermite system. a) Flame radiation characteristics of n‐ABF, μ‐ABF and n‐ABO. b) Binarization process of flame image based on high‐speed photography color images. c) Typical flame area image sequence and d) flame area–time curve. e) Average flame growth rate for each nano‐thermite system.

High‐speed photography was utilized to record the ignition and combustion processes of various nano‐thermite systems under open conditions. The combustion behavior of n‐ABF, μ‐ABO, and n‐ABO systems was analyzed. Typical time‐series images of the ignition process are shown in **Figure** **4**, with all samples exhibiting rapid combustion reactions upon ignition. The ignition and combustion processes for the n‐ABF and n‐ABO were completed within ≈240 µs, while the duration for the µ‐ABF system was within 5 ms. This indicates that the n‐ABF and n‐ABO systems display similar kinetic behavior, whereas the energy release process for the µ‐ABF system is prolonged, highlighting the significant impact of size effects on the ignition and combustion of nano‐thermite systems. In addition, significant differences in combustion and flame radiation phenomena were observed between the systems. **Figure** **5**a illustrates the typical radiative characteristics of the flames in each system. The flame in the n‐ABF system transitions from a bright yellow center to a deep orange periphery, whereas the n‐ABO system exhibits a bright white center with orange edges. The variation in radiative features is attributed to the differences in the aluminum fluorination/oxidation products. The difference in flame brightness among the μ‐ABF, n‐ABF, and n‐ABO systems is primarily attributed to the differences in combustion products and their emissivity. In the n‐ABF system, the primary reaction pathway for aluminum is fluorination, producing AlF₃ as the main product. AlF₃ exists in a gaseous state at high temperatures due to its low boiling points. Condensed phase materials typically exhibit lower energy level transition restrictions and higher densities, which endow them with elevated emissivity in the visible light spectrum. In contrast, the emissivity of gaseous materials is considerably low. Consequently, flames predominantly comprised of gaseous AlF₃ appear relatively dim, whereas flames characterized by condensed phase Al_2_O_3_ appear noticeably brighter. For the μ‐ABF system, the incomplete reaction between μ‐BiF_3_ and n‐Al due to mass transfer limitations results in unreacted n‐Al. This aluminum subsequently reacts with atmospheric oxygen to form Al_2_O_3_, which exists in a condensed state and has a high emissivity. In contrast, the n‐ABF system predominantly forms gaseous AlF_3_ during combustion, which has a much lower emissivity compared to condensed‐phase Al_2_O_3_, leading to a lower flame brightness despite its higher heat release. These findings are consistent with a previous report by Zachariah et al.,^[^ ^16^ ^]^ where more fluorinated products resulted in lower flame temperatures due to differences in product emissivity.

In the μ‐ABF system, the flame edge exhibits more obvious particle characteristics, especially under the condition of *Φ* = 2, where significant high‐temperature particle splashing occurs. This phenomenon indicates that some solid particles are expelled from the reaction zone before the complete reaction. It is generally believed that this phenomenon is caused by the injection of luminous particles separated from the main combustion front, indicating that the reaction between the fuel and the oxidant during the combustion process is incomplete. This incomplete reaction is mainly caused by two reasons: 1) Mass and heat transfer limitations of micron‐scale BiF₃ particles. The large particle size limits the reaction rate between n‐Al and the BiF₃ oxidizer, resulting in incomplete reactions and the formation of unburned particles (Figure S1, Supporting Information). This slow reaction kinetics and uneven reaction propagation contribute to the observed particle splashing phenomenon; 2) Excess fuel under fuel‐rich conditions. Under fuel‐rich conditions, the excess n‐Al cannot fully react with the μ‐BiF₃ or with oxygen in the air. As a result, unburned aluminum particles are expelled from the reaction zone due to the rapid gas generation and the ejection of reaction products during combustion. These unreacted particles are responsible for the high‐temperature particle splashing observed at *Φ* = 2.

To quantitatively assess the kinetic differences among various samples during the ignition process, an analysis was conducted by evaluating the change in flame area over time. The typical image processing procedure is illustrated in Figure 5b, where color images from high‐speed photography are first converted to grayscale and then binarized. To exclude the influence of discrete particles on the flame area, we analyzed the flame area using the maximum connected region in the binarized image. A typical image processing sequence is shown in Figure 5c. Changes in the flame area are analyzed through the variation of the largest connected area. To quantify the differences in the ignition process of each thermite system, the growth rate of the flame reaching its maximum area, denoted as *V_A_*, is used to evaluate the flame growth rate of each system. *V_A_* is calculated according to Equation (1), where *t_max_* represents the time (ms) taken to reach the maximum flame area, and *A_max_* is the maximum flame area (mm^2^). A typical flame area–time curve is depicted in Figure 5d. This method allows for a detailed and quantitative comparison of the ignition kinetics across different compositions.

(1) $$V_{A}=\frac{A_{max}}{t_{max}}\;$$

Figure 5e displays the average flame growth rates for the samples, with n‐ABF showing average rates of 4012, 8588, 9538, and 8941 mm^2^ ms⁻¹ under conditions of *Φ* = 0.75 to 2, respectively. In contrast, the μ‐ABF system exhibits average flame growth rates of 704, 1189, 1935, and 1427 mm^2^ ms⁻¹ under the same conditions. At *Φ* = 0.75 to 2, the average flame growth rates of n‐ABF are ≈4.7 to 6.2 times higher than those of μ‐ABF, indicating significantly superior kinetic characteristics of the n‐ABF system during ignition. This superiority is primarily attributed to the high reactive interface afforded by the nanoscale size of the oxidizer in the n‐ABF system, which promotes reactivity with n‐Al and enhances the system's kinetic properties. In contrast, the reaction between micron‐sized BiF_3_ and n‐Al in the μ‐ABF system has a lower efficiency of mass transfer on the micro‐level, resulting in a reduced rate of flame area growth. This indicates that controlling the microscale of BiF_3_ is an effective method for regulating the energy output rate. Meanwhile, the flame growth rate of the n‐ABO system is slightly lower than that of the n‐ABF system but several times higher than that of the μ‐ABF system. This difference can be attributed to the primary reaction product of Al in the n‐ABF system being AlF_3_, which has a boiling point (1291 °C) significantly lower than the oxidation product of Al (Al_2_O_3_, 2980 °C), making the n‐ABF more prone to producing gaseous flames compared to the n‐ABO system. Additionally, due to fluorine's higher electronegativity compared to oxygen, n‐BiF_3_ is more readily able to gain electrons during combustion reactions than n‐Bi_2_O_3_, resulting in the n‐ABF system having stronger energy release characteristics. The higher flame growth rates of the n‐ABO system compared to the μ‐ABF system by several orders of magnitude underscore that kinetic characteristics in the studied systems are significantly dependent on size effects. Despite fluorine's higher electronegativity, the efficiency of the reaction between n‐Bi_2_O_3_ and n‐Al in combustion reactions far surpasses that of μ‐BiF_3_.

### 2.4 Flame Propagation under Confined Conditions

**Figure 6:**
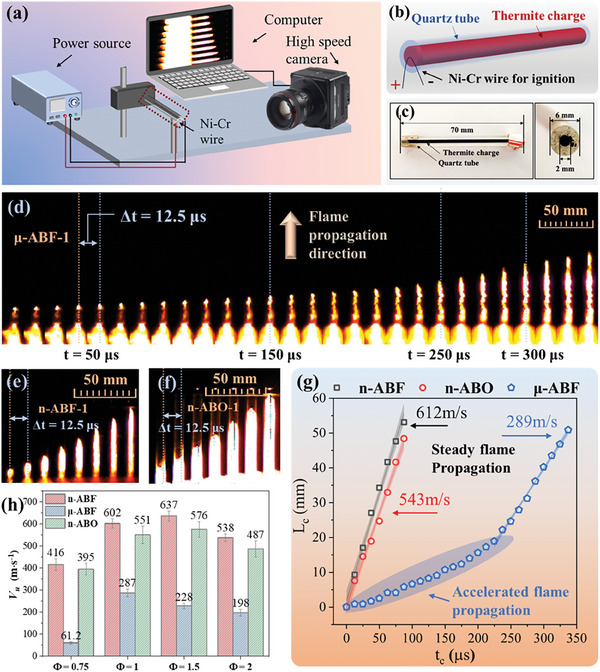
Flame propagation characteristics under confined conditions. a) Schematic diagram of flame propagation characteristics test under constrained conditions. b,c) Schematic diagram and digital photos of constrained conditions based on quartz tubes. High‐speed photography images of flame propagation of d) μ‐ABF‐1, e) n‐ABF‐1, and f) n‐ABO‐1. g) Displacement–time curve of n‐ABF‐1, μ‐ABF‐1 and n‐ABO‐1; h) Flame propagation rate of each nano‐thermite system.

The quartz tube device was used to study flame propagation behavior under confined conditions. This experimental configuration **Figure** **6**a–c simulates an environment where flame extension is spatially restricted, such as a MEMS safety mechanism or a micro‐detonator. The quartz tube restricts gas expansion, resulting in increased pressure and heat accumulation within the system, which significantly affects the flame propagation speed and combustion intensity. During the experiment, the sample powder was loaded into the quartz tube according to the packing density shown in Table S1 (Supporting Information). Figure 6b,c shows a schematic diagram and digital photos. A Ni–Cr heating wire was used to ignite the sample from one end of the quartz tube, and high‐speed photography was used to capture the flame propagation process.

The relationship between flame propagation distance and time was analyzed to evaluate the flame propagation stages and velocities, with time‐resolved high‐speed photographic images under confinement shown in Figure 6d–f. After ignition at one end of the quartz tube, the flame propagated rapidly within the tube. The propagation duration for n‐ABF and n‐ABO was within 100 µs, while for μ‐ABF, the corresponding duration extended to 350 µs. By tracking the positions during the propagation process, the typical displacement‐time curves for n‐ABF, μ‐ABF, and n‐ABO (*Φ* = 1) under confinement are presented in Figure 6g. The curves indicate that μ‐ABF experiences an accelerated flame propagation phase lasting ≈225 µs before transitioning to a stable propagation phase. In contrast, n‐ABF and n‐ABO immediately enter a stable propagation phase. Their acceleration phases are exceedingly brief, falling below the 12.5 µs recording interval of the high‐speed photographic. As a result, from an observational standpoint, flame propagation appears to be instantaneous and continuous, maintaining a constant high velocity. This rapid propagation is likely attributed to confinement, which restricts the diffusion of combustion products and heat, thus concentrating the flame propagation within the enclosed space.^[^ ^30^ ^]^ The combustion reaction generates significant quantities of gases and heat, leading to a swift pressure increase within the enclosed space, thereby granting the nano‐thermite systems under confinement a higher rate of energy release.^[^ ^31^, ^32^ ^]^ In the n‐ABF and n‐ABO systems, a high surface area enhances the contact interface between the fuel and oxidizer, significantly accelerating the chemical reactions essential for the combustion process. This characteristic facilitates the initiation of flame propagation in these systems. Conversely, in the μ‐ABF system, the larger micron‐sized BiF_3_ particles elevate thermal and mechanical resistance during flame propagation, intensifying heat and mass transfer limitations.^[^ ^33^, ^34^ ^]^ Consequently, the μ‐ABF system endures a prolonged acceleration phase before attaining a stable flame propagation stage.

The velocity during the stable flame propagation phase is utilized to evaluate the rate of energy release under confinement, with the calculated results for each nano‐thermite system depicted in Figure 6h. The highest flame propagation velocities were all achieved at *Φ* = 1.5, indicating that the optimal rate of energy release for nano‐thermite systems is slightly below fuel‐rich conditions. The flame propagation speeds of n‐ABF and n‐ABO are ≈1 to 2 times higher than that of μ‐ABF. This trend aligns with previous results regarding flame growth, further emphasizing the significance of size effects within nano‐thermite systems. The flame propagation velocity of n‐ABF exceeds that of n‐ABO by 53 to 199 m s^−^¹. This phenomenon is likely due to the primary reaction product in the n‐ABF system being AlF_3_, rather than Al_2_O_3_. This implies that the n‐ABF system generates a greater quantity of gaseous products, which can facilitate thermal convection during the reaction and increase pressure accumulation under confinement.^[^ ^35^, ^36^]

### 2.5 Flame Propagation under Unrestrained Conditions

**Figure 7:**
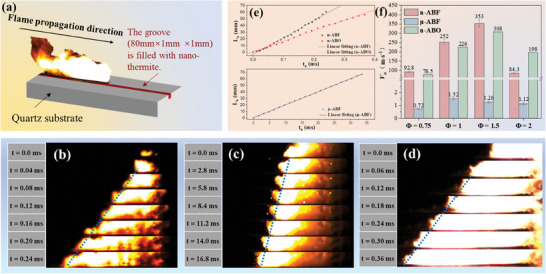
Flame propagation characteristics under unrestrained conditions. a) A schematic diagram of a flame spread test under open conditions. b–d) High‐speed photographic time series images of flame spread for n‐ABF, μ‐ABF, and n‐ABO, and the corresponding e) displacement–time curves. f) Average flame spread rate for different nano‐thermite systems.

The flame propagation properties of energetic materials in an open environment were investigated by placing the sample in a quartz container with a slotted cavity, as depicted in **Figure** **7**a. This experimental setup allows the flame to propagate without spatial restrictions, providing ideal conditions for studying flame dynamics in free space.

Figure 7b–d displays the time‐series high‐speed photographic images of flame propagation for each nano‐thermite system, showing rapid flame spread upon ignition, with simultaneous propagation of the reaction front and combustion toward the gaseous phase. Figure 7e presents the relationship curve between the flame front and time captured by high‐speed photography. The linear relationship between displacement and time for flame propagation under open conditions indicates uniform flame spread in open conditions. Figure 7f reveals the flame propagation rates for each system, with the maximum flame propagation rates for all systems being achieved at an equivalence ratio of 1.5. The flame propagation speed of the n‐ABF system is 9.1 to 45.0 m s⁻¹ higher than that of the n‐ABO system. Notably, flame propagation under confinement exceeds that under open conditions for all nano‐thermite systems. The values for μ‐ABF under confinement surpass those under open conditions by one to two orders of magnitude, while for n‐ABF and n‐ABO systems, the increase is only by several folds (1 to 6 times). This indicates that the effect of confinement on the n‐ABF and n‐ABO systems is less significant than on the μ‐ABF system.

### 2.6 Ignition Temperature

**Figure 8:**
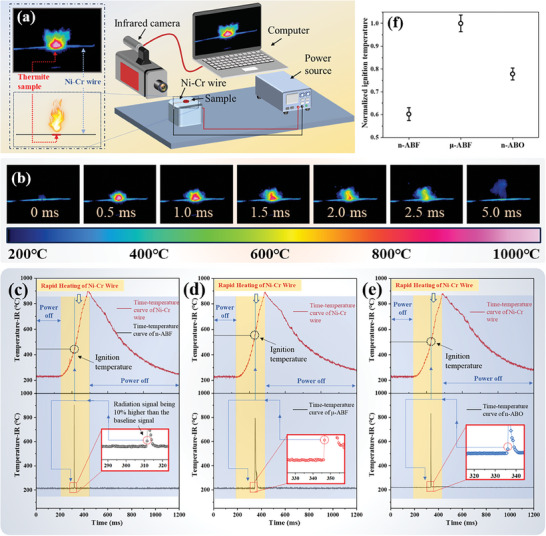
Comparison of ignition temperatures of various nano‐thermite systems. a) Schematic diagram of ignition temperature measurement. b) Infrared thermographic image of a typical ignition process (μ‐ABF‐1). c–e) Typical infrared temperature curves of the Ni‐Cr wire and the samples n‐ABF, μ‐ABF, and n‐ABO. f) Normalized ignition temperature.

To assess the differences in ignition temperatures among various nano‐thermite systems, infrared thermography, and a rapidly heating Ni‐Cr wire were used to test the ignition temperatures of n‐ABF, n‐ABO, and μ‐ABF. The measurement method for ignition temperatures is based on the approach by Dreizin et al.^[^ ^37^ ^]^ and prior research,^[^ ^18^ ^]^ which involves synchronously monitoring the infrared temperatures of both the nickel‐chromium wire and the sample portion during the ignition process. A schematic diagram of the testing setup is shown in **Figure** **8**a, where nano‐thermite samples are placed in a small tray through which the Ni‐Cr wire passes. While the electric heating wire is heated by a direct current source, the entire ignition process is monitored using a high‐speed infrared thermal imager. The temperature of the Ni‐Cr wire at the moment the nano‐thermite sample just begins to ignite, indicated by the emission of a radiation signal, is taken as the ignition temperature. Figure 8b displays typical time‐resolved images captured by the high‐speed infrared camera of the μ‐ABF ignition process. As current passes through the nickel‐chromium wire, the temperature rapidly increases until the combustion reaction occurs upon reaching the sample's ignition temperature. Figure 8c–e presents the infrared temperature curves for the Ni–Cr wire (red curve in the upper part) and the various nano‐thermite systems (black curves in the lower part) during the experiment. The moment of sample ignition is determined by the black curve, with the corresponding temperature of the Ni‐Cr wire at that moment, as indicated by the red curve, representing the ignition temperature.

Given that the temperature obtained from infrared thermometry depends on the emissivity of the surface being measured, it presents a challenge. Research has shown that the emissivity of Ni‐Cr wire changes during the heating process due to variations in temperature and the inevitable degree of oxidation.^[^ ^38^ ^]^ Consequently, this study employs normalized infrared radiation intensity based on the Stefan–Boltzmann law to evaluate ignition temperatures, taking into account the optical characteristics of infrared thermometry. The emissivity of Ni–Cr wire during high‐temperature heating is difficult to ascertain, necessitating the use of an infrared thermal imager to measure the “temperature” of the Ni–Cr wire, which essentially reflects the wire's “radiative temperature” based on radiation intensity. Nonetheless, “radiative temperature” can still be utilized to distinguish between the ignition temperature differences among various samples.

Figure 8f shows the normalized ignition temperatures. The μ‐ABF system has the highest ignition temperature, followed by the n‐ABO system. The n‐ABF system exhibits the lowest ignition temperature. The highest ignition temperature in the μ‐ABF system indicates that the size effect is predominant in influencing the ignition threshold of nano‐thermite systems. The n‐ABF and n‐ABO systems, leveraging nanoscale oxidizers, possess a higher reactive interface, resulting in lower ignition temperatures compared to the μ‐ABF system. The ignition temperature of the n‐ABF system is lower than that of the n‐ABO system. This phenomenon can be ascribed to the higher electronegativity of fluorine compared to oxygen, which confers upon bismuth fluoride a more potent electron‐accepting capacity than bismuth oxide.

### 2.7 Combustion Temperature

Combustion temperature is one of the important characteristic parameters of the combustion process. Multiwavelength thermometry^[^ ^39^ ^]^ was used to evaluate combustion temperatures. The method is based on Planck's law (Equation (2)).

(2) $$E\;\left(T,\lambda \right)=\varepsilon \cdot \frac{c_{1}}{\lambda^{5\left[\exp\left(c_{2}/\lambda T\right)-1\right]}}$$

where the emission intensity of thermal radiation is a function of surface emissivity (*ε*), wavelength (*λ*), and temperature (*T*), and *c*_1_ and *c*_2_ are Planck's constants. The researchers found that in the combustion of nano‐thermite systems, it is necessary to assume a functional dependence of emissivity (*ε* as a function of *λ*) in the calculation to calculate the temperature, as shown in Equation (3). Formula (4) is obtained by taking the natural logarithm on both sides of formula (2). Substituting the assumed ε wavelength function into Equation (4) gives *Z*, defined by Equation (5). Use a plot of *Z* versus the reciprocal wavelength to determine the temperature of the slope.

(3) $$\varepsilon \;\left(\lambda \right)=\frac{C_{3}}{\lambda }\;$$

(4) $$\ln\left(\varepsilon \left(\lambda ,T\right)\times \frac{C_{1}}{\lambda^{5}}\times {\left[E\left(T,\lambda \right)\right]}^{-1}\right)=\frac{C_{2}}{\lambda T}\;$$

(5) $$Z=\ln\left(\frac{C_{1}}{\lambda^{6}}\times {\left[E\left(T,\lambda \right)\right]}^{-1}\right)=\frac{C_{2}}{\lambda T}\;-\ln\left(C_{3}\right)$$

**Figure 9:**
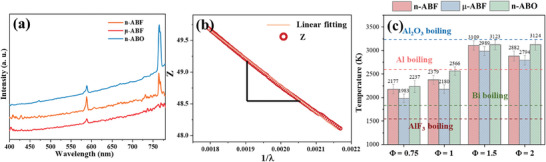
Combustion temperatures of various nano‐thermite systems based on emission spectroscopy. a) Typical emission spectrum curve; b) Linear fitting of *Z* − 1/*λ*; c) Combustion temperature of each nano‐thermite system.

**Figure** **9**a showcases the typical emission spectra of the systems, all of which exhibit characteristic blackbody radiation features. Notably, both the n‐ABF and µ‐ABF systems display strong emission features of Na and K atoms. It is important to highlight that neither the n‐ABF nor the µ‐ABF systems detected emission features related to AlO and AlO_2_, which may be attributed to the fluorides in the system's oxidizers acting as “oxidants” and the higher electronegativity of fluorine suppressing the reaction between n‐Al and atmospheric oxygen during the process. Based on a linear fit of the emission spectra according to Equation (5) (Figure 9b), the calculated combustion temperatures are presented in Figure 9c. The combustion temperatures for n‐ABF range between 2177 and 3109 K, ≈120 to 194 K higher than those for µ‐ABF (1983 to 2989 K). The combustion temperatures of the n‐Al/BiF_3_ system are higher than the boiling points of its main combustion products, Bi and AlF_3_, but lower than the boiling point of aluminum. Notably, the boiling points of all theoretical combustion products in the n‐Al/BiF_3_ system are lower than its combustion temperatures. The formation of gaseous products facilitates thermal convection within the reaction system, enhancing the system's energy release performance. The combustion temperatures for n‐ABO (2237 to 3124 K) are ≈100 K higher than those for n‐ABF and μ‐ABF. The combustion temperature of the n‐Al/Bi_2_O_3_ system exceeds the boiling point of its primary combustion product, Bi, yet remains below the boiling point of Al_2_O_3_. This indicates that bismuth inorganic fluoride (BiF_3_), in comparison to its oxide (Bi_2_O_3_), possesses the potential to offer enhanced gas generation capabilities in the construction of MICs.

### 2.8 Pressurization in a Constant‐Volume Container

**Figure 10:**
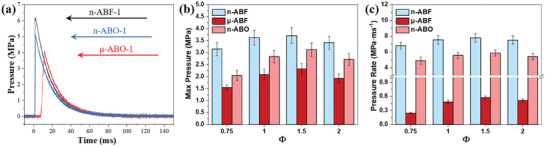
The pressurization capability of each nano‐thermite system. a) Pressure–time curves and b) peak pressure versus c) pressurization rate for each nano‐thermite system.

An enclosed bomb calorimeter equipped with a pressure sensor was employed to evaluate the pressure output characteristics of various nano‐thermite reaction systems. By assessing the pressure–time curves in **Figure** **10**a, the peak pressures and pressurization rates of each sample were determined. The peak pressure is primarily related to the total volume of gases produced and the thermal expansion of gases during the reaction, while the pressurization rate is determined by the slope of the ascending segment of the pressure–time curve. As shown in Figure 10b,c, n‐ABF has the strongest pressure output capacity. At various *Φ*, its peak pressure and pressurization rate are 1.6–2.0 times and 13.6–28.3 times that of μ‐ABF, respectively. This difference is due to the more complete chemical reaction in the n‐ABF system during the reaction. The nano‐scale fuel and oxidant in n‐ABF have a larger reaction interface and more thorough chemical conversion, resulting in a more comprehensive release of energy and substances during the reaction. The peak pressure and pressurization rate of n‐ABF are also slightly higher than those of n‐ABO. This phenomenon can be attributed to the formation of a larger volume of low‐boiling point gaseous aluminum fluoride in the n‐ABF reaction, thereby improving the pressure output capacity of the system. In addition, the best pressure output performance of each system is achieved at *Φ* = 1.5, which is consistent with the previous trend of energy release.

### 2.9 Reaction Mechanism

**Figure 11:**
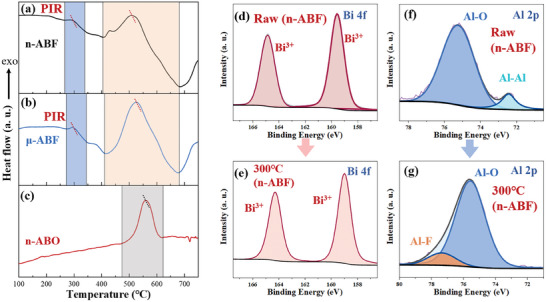
Analysis of thermal reaction paths of various nano‐thermite systems. a–c) DSC curves of n‐ABF, μ‐ABF, and n‐ABO; Bi 4f high‐resolution spectrum of d) the original sample and e) sample at 300 °C; Al 2p high‐resolution spectrum of f) the original sample and g) sample at 300 °C.

In order to further study the chemical reaction mechanism of the BiF_3_‐based nano‐thermite system, the thermal analysis technique was used in combination with the combustion products to analyze the thermal reaction path. **Figure** **11**a–c presents the thermal analysis curves for the n‐ABF, μ‐ABF, and n‐ABO systems. Under controlled temperature ramp conditions, n‐ABF and μ‐ABF exhibited similar thermal reaction characteristics. The DSC curve of n‐ABF Figure 11a displayed two distinct exothermic peaks. The first exothermic peak, located in the temperature range of 262 to 328 °C with a smaller peak area, reflects the PIR between n‐Al and n‐BiF_3_.^[^ ^20^, ^40^, ^41^ ^]^ The second exothermic peak, appearing in the temperature range of 412 to 671 °C, represents the system's main exothermic reaction. The thermal reaction pathway of the μ‐ABF's DSC curve is similar to that of n‐ABF, but the temperature range of the first exothermic peak is at 276 to 344 °C, ≈14 °C higher than the corresponding peak in the n‐ABF system. The temperature range of the second exothermic peak is similar to that of n‐ABF. In contrast, the PIR temperature of n‐ABF is lower. The lower PIR temperature indicates that the reaction between n‐Al and n‐BiF is more readily initiated, with tighter interface contact, benefiting the overall combustion performance and reaction rate. In Figure 11c, the curve for n‐ABO exhibits a significant exothermic peak at a peak temperature of 557 °C, indicating a typical condensed phase exothermic reaction between n‐Al and Bi_2_O_3_ in the metal oxide‐based nano‐thermite system.^[^ ^42^, ^43^, ^44^ ^]^

Under rapid reaction conditions, n‐ABF's reactivity is significantly higher than μ‐ABF's. However, under controlled temperature ramping, both systems exhibit similar thermal reaction pathways. This may be because the reaction under programmed heating conditions is slower, allowing for ample mass and heat transfer between the fuel and oxidizer within the systems. Therefore, n‐ABF and μ‐ABF demonstrate similar thermal reaction pathways. The exothermic temperature of the n‐ABO system is lower than that of the BiF_3_‐based nano‐thermite systems, attributable to two factors: on one hand, the PIR between the high electronegativity fluorine elements and the Al_2_O_3_ shell of n‐Al promotes the exposure of highly reactive aluminum cores; on the other hand, due to fluorine's higher electronegativity compared to oxygen, the system using BiF_3_ as the oxidizer more readily extracts electrons from n‐Al, thus undergoing an exothermic reaction.

Figure 13d–g displays the XPS spectra of n‐ABF material in its pristine state and after heating to 300 °C. The high‐resolution Bi 4f spectra for pristine n‐Al/BiF_3_ and at 300 °C both exhibit pronounced spin–orbit splitting peaks corresponding to trivalent Bi, particularly Bi 4f 7/2 and Bi 4f 5/2.^[^ ^45^, ^46^ ^]^ The reaction at 300 °C predominantly occurs at the interface of the Al_2_O_3_ shell rather than at the interface with the aluminum core. The high‐resolution Al 2p spectra of the pristine n‐Al/BiF_3_ sample reveal characteristic peaks corresponding to Al–O and Al–Al, associated respectively with the oxidized shell and aluminum core of the Al NPs.^[^ ^44^ ^]^ In the high‐resolution Al 2p spectra of the n‐Al/BiF_3_ system at 300 °C, characteristic peaks corresponding to Al–O and Al–F are observed,^[^ ^18^ ^]^ indicating that the n‐Al/BiF_3_ system undergoes a PIR process (Equation (6)).

(6) $$Al_{2}O_{3}+\mathrm{Bi}F_{3}\to \mathrm{Al}O_{x}F_{y}+\mathrm{Bi}F_{x}O_{y}$$

**Figure 12:**
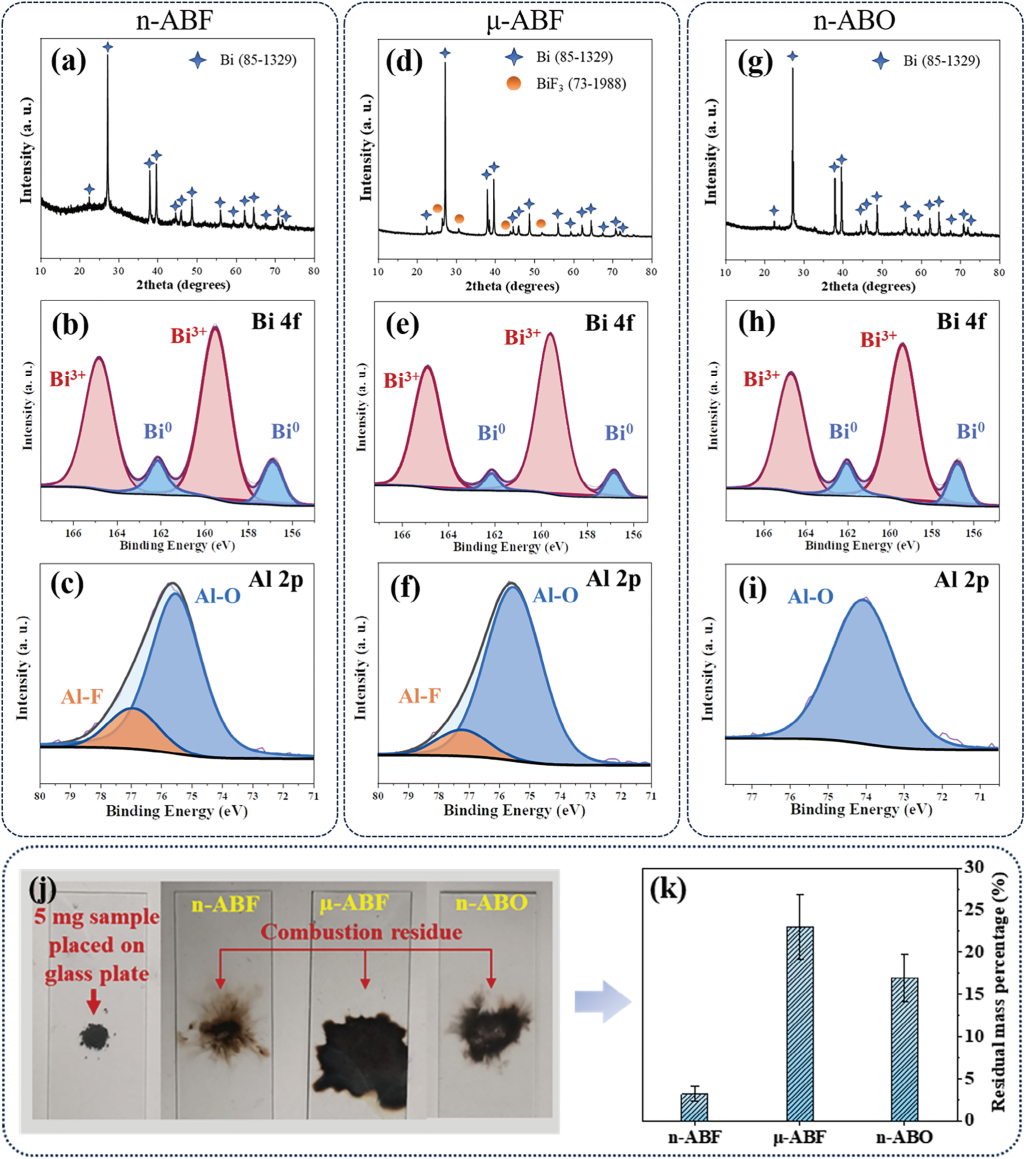
Analysis of combustion products of each nano‐thermite system. XRD and XPS characterization analysis of combustion products based on a–c) n‐ABF, d–f) μ‐ABF and g–i) n‐ABO. j) Digital image and residual mass percentage of each nano‐thermite system after combustion.

XRD and XPS analyses were conducted to characterize the combustion products, with **Figure** **12** showcasing the corresponding results. Figure 12a–c displays the XRD spectra and high‐resolution XPS spectra of the combustion products from the n‐ABF system. The XRD spectra reveal diffraction peaks corresponding to Bi (JCPDS No.85‐1329), indicating that the reduction product of BiF_3_ is metallic Bi. This is corroborated by the XPS results, as shown in Figure 12b with high‐resolution Bi 4f spectra exhibiting characteristic peaks at binding energies of 159.14 and 156.84 eV, corresponding to metallic Bi in the zero valence state.^[^ ^46^ ^]^ Notably, the XRD results did not reveal any diffraction peaks related to Al combustion products, consistent with phenomena observed in our previous research,^[^ ^18^ ^]^ possibly due to relevant products not necessarily existing in sufficiently high concentrations or crystallinities to be detected. Thus, it's necessary to utilize XPS to characterize combustion products related to Al. The high‐resolution XPS Al 2p spectrum (Figure 12c) at a binding energy of 76.98 eV corresponds to Al–F, indicating that Al combines with F from BiF_3_ during the combustion reaction, releasing energy. The peak at a binding energy of 75.58 eV corresponds to Al–O, originating from the passivating oxide shell of unreacted nanoscale Al.

Figure 12d–f presents the corresponding characterization results for the combustion products of the μ‐ABF system. The XRD spectrum (Figure 12d) not only displays diffraction peaks corresponding to Bi but also to BiF_3_ (JCPDS No.73‐1988). The characteristics of the high‐resolution XPS Al 2p and Bi 4f spectra are similar to those corresponding to the n‐ABF system, indicating that in the μ‐ABF reaction, besides the fluorination of Al and reduction of Bi to its elemental form, some BiF_3_ did not participate in the reaction, implying an incomplete reaction. This may be due to the size of BiF_3_ in the μ‐ABF system being an order of magnitude larger than n‐Al, leading to a slower interfacial mass transfer process during the combustion reaction, thereby resulting in incomplete system reactions. Figure 12g–i showcases the corresponding characterization results for the combustion products of the n‐ABO system. The XRD spectrum (Figure 12g) reveals diffraction peaks corresponding to Bi, indicating that the reduction product of Bi_2_O_3_ in the system is metallic Bi. The high‐resolution XPS Bi 4f spectrum in Figure 12h corroborates this result with characteristic peaks corresponding to metallic Bi in the zero oxidation state. The high‐resolution XPS Al 2p spectrum in Figure 12i at a binding energy of 74.08 eV corresponds to Al–O, originating from aluminum's oxidation products.

Beyond the chemical characterization of the combustion products, their macroscopic state also serves as an important reference for reflecting the combustion kinetics characteristics of the systems. Figure 12j displays digital images of the combustion products. Before testing, 5 mg of nano‐thermite samples were placed on a glass plate. After ignition, all samples left corresponding combustion products on the glass plate. The n‐ABF system left a thin layer of dark residue on the glass plate, with the lightest color and smallest area among all systems. The n‐ABO system's combustion products were thicker and darker in color, while μ‐ABF left behind the largest area of combustion products, also the darkest in color. To quantify the residue rates of each system, experiments were conducted using the mass of residue left after igniting 100 mg of samples. As shown in Figure 12k, the residue rates for the n‐ABF, μ‐ABF, and n‐ABO systems are ≈3%, 23%, and 17%, respectively. The lowest residue rate of n‐ABF confirms previous speculations, illustrating the characteristic of the nano‐thermite system constructed with nanoscale bismuth fluoride as the oxidizer, namely that its combustion products almost entirely leave the reaction zone in the gaseous form. The higher residual rate of μ‐ABF also emphasizes the importance of the size effect in the reaction completeness of the nano‐thermite system.

In the Al/Bi_2_O_3_ system, the initiation of the reaction occurs in the condensed phase. Al acts as a reducing agent to capture O^2−^ in Bi_2_O_3_ for reaction. This process precedes the decomposition of Bi_2_O_3_ and the release of O_2_. This reaction process produces Al_2_O_3_ with a high melting point and reduces Bi_2_O_3_ to metallic Bi with a boiling point lower than the combustion temperature. It exhibits an exothermic reaction characteristic that is self‐sustaining and rapidly propagates under high‐temperature conditions. In addition, unlike transition metal oxides such as CuO, Fe_2_O_3_, and MoO_3_ that are common in nano‐thermite systems, metal Bi, the reduction product of Bi_2_O_3_, leaves the reaction interface in the form of a gaseous state rather than a condensed state during combustion. This feature effectively promotes the transfer of reaction heat from the reaction zone to the unreacted zone, further enhancing the efficiency and diffusion of the reaction.

**Figure 13:**
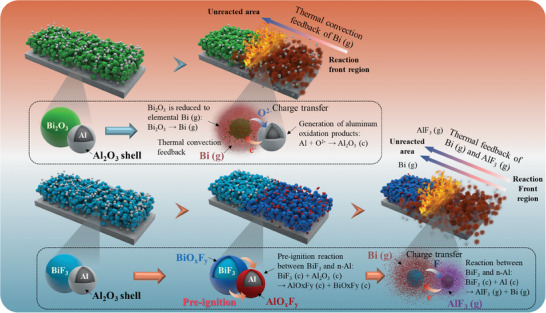
Schematic diagram of the reaction mechanism of Al/BiF_3_ and Al/Bi_2_O_3_ systems.

In the context of n‐Al, two primary challenges exist regarding energy release during reactions: first, the formation of an oxide shell in the presence of air elevates the ignition threshold; second, the production of high‐melting‐point oxidation products during the reaction impedes their departure from the reaction interface, leading to reactive sintering. Within the Al/BiF_3_ system, a PIR occurs before reaching the ignition temperature, which corrodes the alumina shell and exposes the active aluminum core. This PIR facilitates the breakdown of the alumina shell and the exposure of the active aluminum surface (Figure S2, Supporting Information). Crucially, in the Al/BiF_3_ system, the low boiling point of both the BiF_3_ reduction product (Bi) and the fluorination product of aluminum (AlF_3_) enables their easy departure from the reaction interface. Additionally, the formation of more gaseous products aids in the transfer of heat from the reaction zone to unreacted areas, mitigating sintering during the reaction and reducing post‐reaction residue (**Figure** **13**).

A comparative analysis of various nano‐thermite systems reveals that the n‐ABF system exhibits superior kinetic performance, including higher pressure output and flame growth rate, as shown in Figure S3 (Supporting Information). This is attributed to the formation of low‐boiling‐point products during combustion, which enhance pressure generation and reduce residue. The n‐ABF system also demonstrates a slightly lower combustion temperature due to the lower emissivity of gaseous products compared to condensed‐phase ones.

As demonstrated by thermal analysis, the thermite reaction occurs in the condensed phase at the interface of aluminum and bismuth fluoride. Both nano‐ and micro‐scale thermite systems exhibit similar thermal reaction pathways, indicating a fundamental consistency in the reaction mechanisms. However, the nanoscale thermite system benefits from the small dimensions of both the oxidizer and fuel, facilitating rapid mass and heat transfer between them. This ease of interaction activates the unreacted region through thermal convection at the reaction front, thereby enhancing the overall reaction kinetics.

In contrast, within the μ‐BiF_3_ system, the reaction initiates at the interface between Al NPs and μ‐BiF_3_. The substantial size difference between the μ‐BiF_3_ and Al NPs, where μ‐BiF_3_ is an order of magnitude larger than the Al NPs, results in an increasing distance between the remaining μ‐BiF_3_ and Al NPs. This separation exacerbates the difficulty of mass and heat transfer between them, leading to a significantly reduced reactivity of the microscale thermite system compared to its nanoscale counterpart. The size effect, therefore, plays a crucial role in determining the reaction kinetics, with the nanoscale system exhibiting superior performance due to enhanced interfacial reactivity and efficient heat and mass transfer.

### 2.10 Evaluation of Interface Electron Transfer Capabilities Based on DFT Calculations

**Figure 14:**
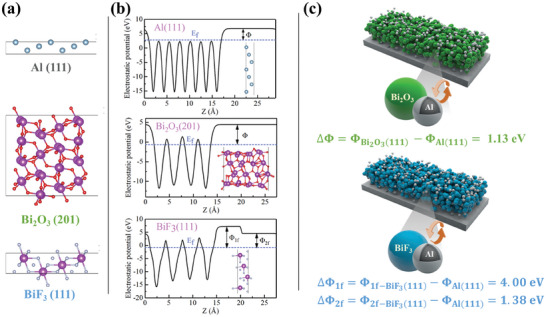
Evaluation of interface electron transfer capabilities. a) Models established by DFT calculations: Al (111), Bi_2_O_3_ (201), and BiF_3_ (111). b) Electrostatic potential distributed along the *Z*‐direction of Al (111), Bi_2_O_3_ (201), and BiF_3_ (111). c) The work function difference between nano‐thermite systems.

Referring to DFT, theoretical first‐principles calculations were performed under the VASP program to further study the difference in energy release between the Al/Bi_2_O_3_ and Al/BiF_3_ systems. The models constructed for the calculations are depicted in **Figure** **14**a, with seven atomic layers for Al (111), four molecular layers for Bi_2_O_3_ (201), and four molecular layers for BiF_3_ (111) thickness. All these slab models are constructed from stoichiometric surfaces. Previous research has shown that the F‐terminated surface in BiF_3_ (111) is more stable.^[^ ^47^ ^]^ In the slab model with F‐terminated exposure, the upper surface exposes one F atom, named 1f‐BiF_3_ (111), and the lower surface exposes two F atoms, named 2f‐BiF_3_(111). For the slab models of these three systems, *Γ‐*centered *k*‐point meshes of 16  ×  16  ×  1 for Al (111), 2  ×  4  ×  1 for Bi_2_O_3_ (201), and 6  ×  6  ×  1 for BiF_3_ (111) were used, respectively. All slab models were designed with a vacuum layer thickness of 15 Å, and calculations considered dipole corrections along the *z*‐direction. The convergence criterion for forces during structural optimization was set at 0.03 eV Å^−1^, and the energy convergence criterion for electronic step iterations was set at 10^−5^ eV.

The intrinsic essence of violent redox reactions in composite energetic systems is the transfer of electrons. When the interface of fuel and oxidizer is in contact, the work function (Φ_wf_) is used to evaluate the flow direction and ability of interface electron transfer:^[^ ^48^ ^]^

(7) $$\Phi_{\mathrm{wf}}=-E_{\mathrm{Fermi}}+E_{\mathrm{vacuum}}$$

In the Equation (7), *E*_vacuum_ and *E*_Fermi_ represent the vacuum electrostatic potential and Fermi level in the slab model respectively. The work function is a measure of the minimum energy required to remove an electron from the solid to a point in the vacuum close to the surface. In the context of thermite reactions, a lower work function indicates a higher tendency of the material to donate electrons, promoting the reduction reaction. Conversely, a higher work function suggests a material acts more readily as an electron acceptor, facilitating the oxidation process. By evaluating the work functions of the interfaces in Al/Bi_2_O_3_ and Al/BiF_3_ systems, one can infer the efficiency and direction of electron transfer, which directly influences the reactivity and energy release of the system.

The 1D Planar‐Average Potential along the *z*‐direction for Al (111), Bi_2_O_3_ (201), 1f‐BiF_3_ (111), and 2f‐BiF_3_ (111) surfaces is illustrated in Figure 14b. The Φ_wf_ for these surfaces are listed in Table S2 (Supporting Information), with Al (111), Bi_2_O_3_ (201), 1f‐BiF_3_ (111), and 2f‐BiF_3_ (111) having work functions of 4.02, 5.15, 8.03, and 5.40 eV, respectively. Among all surfaces, Al (111) has the lowest work function, indicating that electrons are likely to transfer from the fuel Al to the oxidizers Bi_2_O_3_ or BiF_3_ upon interface formation to achieve a common Fermi level due to the different Fermi energy levels of the surfaces, aligning with the actual redox reaction scenario.

Furthermore, the difference in work function between Al (111) and Bi_2_O_3_ (201) is 1.13 eV, which is smaller than the differences between Al (111) and 1f‐BiF_3_ (111) of 4.00 eV and between Al (111) and 2f‐BiF_3_ (111) of 1.38 eV (Figure 14c). This implies that the electron transfer driving force between Al and BiF_3_ is stronger, making redox reactions more likely to occur. This observation corroborates experimental findings where the Al/BiF_3_ system exhibits a higher reaction rate and lower ignition temperature compared to the Al/Bi_2_O_3_ system. The significant work function difference, especially between Al (111) and 1f‐BiF_3_ (111), suggests a high propensity for electron transfer, contributing to the enhanced reactivity and energetic performance of the Al/BiF_3_ system.

## 3 Conclusion

The research on the reaction kinetics of MICs based on metal fluorides has provided valuable insights into the combustion behavior and reactivity of these advanced materials. By incorporating BiF_3_ as an oxidant in a nano‐thermite system, the study has demonstrated significant advancements in terms of energy release, reactivity, and combustion characteristics. The experimental data revealed that the n‐Al/n‐BiF_3_ system exhibited notable improvements in reactive kinetic properties compared to the traditional system (n‐Al/n‐Bi_2_O_3_). The system displayed a swift flame expansion rate of 8588 mm^2^ s^−1^ and a deflagration wave propagation velocity of 602 m s^−1^, indicating enhanced reactivity and energy release capabilities. Moreover, the peak pressure (3.62 MPa) and pressure escalation rate (7.54 MPa s^−1^) of the n‐Al/n‐BiF_3_ system surpassed those of the n‐Al/n‐Bi_2_O_3_ system by 27.9%, underscoring the superior performance of the metal fluoride‐based energetic composite. The virtually non‐existent condensed‐phase combustion products, facilitated by the low boiling points of Bi and AlF_3_, eliminated agglomeration issues and prevented the entrapment of active metals. This smooth conduction of heat flux contributed to the efficient reaction kinetics and enhanced reactivity of the system. Detailed analysis of the reaction mechanism through thermal analysis and product characterization techniques identified the PIR mechanism within the n‐Al/BiF_3_ system, where the highly electronegative fluorine in BiF_3_ played a crucial role in corroding the Al_2_O_3_ shell on Al NPs. This RIP process effectively reduced the ignition threshold of the system, enabling quicker and more efficient ignition compared to conventional systems. The DFT calculations reveal that the work function disparity between the Al (111) surface and the Bi_2_O_3_ (201) surface is 1.13 eV. This value is significantly smaller than the disparities observed between Al (111) and 1f‐BiF_3_ (111) at 4.00 eV and between Al (111) and 2f‐BiF_3_ (111) at 1.38 eV. This reduced work function difference implies a more pronounced propensity for electron transfer at the Al/BiF_3_ interface, thereby facilitating redox reactions. These computational insights are substantiated by experimental findings, which demonstrate that the n‐Al/n‐BiF_3_ system possesses both a more rapid reaction rate and a lower ignition temperature than the n‐Al/n‐Bi_2_O_3_ system. The significance of this research lies in its contribution to the development of high‐performance energetic materials with ultra‐efficient reactivity and virtually non‐existent condensed phase products. This advancement positions it as a promising candidate for n‐Al‐based reactive materials in the fields of propellants, explosives, and pyrotechnics.

## 4 Experimental Section

### Materials

Al NPs (average particle size 70 nm) were acquired from Shanghai Bicon New Material Technology Co., Ltd., with an active aluminum content of ≈71%, as determined by thermogravimetric analysis (TGA) under atmospheric conditions (Figure S4, Supporting Information). Bismuth nitrate pentahydrate (Analytical Reagent, AR), ammonium fluoride (AR), and sodium borofluoride (AR) were sourced from Macklin. Ethylene glycol (AR), *n*‐hexane (AR), and anhydrous ethanol (AR) were purchased from Sinopharm Chemical Reagent Co., Ltd. The average particle size of nano‐bismuth oxide is 220 nm (Figure S5, Supporting Information), which was purchased from Shanghai Chaowei Nanotechnology Co., Ltd.

### Synthesis of Bismuth Trifluoride and Preparation of Nano‐Thermite System

**Figure 15:**
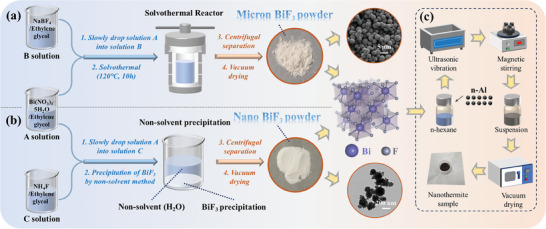
Typical synthesis process of a) n‐BiF_3_ and b) μ‐BiF_3_; c) Ultrasonic mixing process of the nano‐thermite system.

**Figure** **15** illustrates a schematic diagram of the synthesis of BiF_3_ and the preparation process of the nano‐thermite system. Both μ‐BiF_3_ and n‐BiF_3_ were synthesized using distinct chemical processes.

μ‐BiF_3_ was synthesized employing the method proposed by Bharat,^[^ ^49^ ^]^ as depicted in Figure 15a. In a typical synthesis procedure, 0.97 g of Bi(NO_3_)_3_·5H_2_O was dissolved in 20 mL of ethylene glycol, referred to as Solution A. Meanwhile, 0.08 g of NaBF_4_ was dissolved in 20 mL of ethylene glycol, forming Solution B. Solutions A and B were magnetically stirred for 10 h until completely dissolved. Subsequently, Solution B was slowly added to Solution A at a rate of 2 mL min^−1^, followed by continuous magnetic stirring for 40 min. The mixed solution was then transferred to a 100 mL polytetrafluoroethylene‐lined autoclave and reacted at 120 °C for 10 h. After cooling the reaction mixture to room temperature, the solid product was collected by centrifugation and washed several times with water and ethanol. The product was then vacuum‐dried overnight at 80 °C, yielding a white μ‐BiF_3_ solid powder.

n‐BiF_3_ was synthesized according to the method proposed by Meng.^[^ ^28^ ^]^ In the typical synthesis protocol (Figure 15b), Solution A was prepared as described above. 0.22 g of NH_4_F was dissolved in 20 mL of ethylene glycol to form Solution C. Both solutions were magnetically stirred for 10 h until fully dissolved. Then, Solution C was slowly added to Solution A at a rate of 2 mL min^−1^, with continuous magnetic stirring for 40 min. Finally, using a nonsolvent precipitation method, an excess of nonsolvent (water) was added to the mixture, which was then left undisturbed after stirring for 10 min. A white precipitate formed at the bottom of the container, which was collected by centrifugation. After vacuum drying overnight at 80 °C, a powdery white n‐BiF_3_ solid was obtained.

The nano‐thermite system was prepared using a classical ultrasonic blending method, as shown in Figure 15c. In a typical preparation process, a certain mass of BiF_3_ and n‐Al were ultrasonically dispersed in *n*‐hexane for 30 min, followed by magnetic stirring for 12 h. Subsequently, the samples were dried in a vacuum oven for 2 h to evaporate the *n*‐hexane. The powders were then gently sieved through a 40‐mesh screen using a brush to break up agglomerates. The specific mixing ratios of the samples are presented in Tables S3 and S4 (Supporting Information). The nano‐thermite systems based on n‐BiF_3_, μ‐BiF_3_, and n‐Bi_2_O_3_ were respectively named n‐ABF, μ‐ABF, and n‐ABO.

The equivalence ratio (*Φ*) is defined by Equation (8), where ACT and ST represent the actual ratio and the stoichiometric ratio, respectively. The stoichiometric reaction between aluminum and oxidizer can be indicated by Equation (9). In the calculation of the actual ratio, the value of active aluminum was used.

(8) $$\Phi =\frac{{\left(\mathrm{Al}/\mathrm{Bi}F_{3}\right)}_{\mathrm{ACT}}}{{\left(\mathrm{Al}/\mathrm{Bi}F_{3}\right)}_{\mathrm{ST}}}\;$$

(9) $$\mathrm{Al}+\mathrm{Bi}F_{3}\to \mathrm{Al}F_{3}+\mathrm{Bi}$$

### Characterization Methods

The crystal structure of the samples was analyzed using an X‐ray diffractometer (XRD, Bruker D8 Advance). The morphology and structure of the samples were characterized using a scanning electron microscope (SEM, FEG 250), and their elemental composition was determined through energy‐dispersive X‐ray spectroscopy (EDS, Oxford Instruments) attached to the SEM. The microstructure of the samples was examined using a transmission electron microscope (TEM, FEI Tecnai). X‐ray photoelectron spectroscopy (XPS) measurements were conducted using a monochromatic Al Kα X‐ray source (1486.6 eV) on a Thermo Scientific ESCALAB 250i system. The chamber pressure was maintained at 10^−10^ mbar and all binding energies were referenced to the C 1s peak at 284.8 eV. Differential Scanning Calorimetry (TGA/DSC 3+, METTLER TOLEDO) was utilized for thermal analysis, with a heating rate of 20 °C min^−1^ and an Ar flow rate of 30.0 mL min^−1^. The particle size distribution of the samples was analyzed using a laser particle size analyzer (Mastersizer 2000/3000, Malvern) during the experimental process.

### Combustion Characterization

To comprehensively investigate the flame propagation behavior of energetic materials under varying environmental constraints, experiments were designed to simulate two typical application scenarios: an open environment and a constrained environment. The open environment was modeled using a quartz container with a strip‐shaped groove chamber, allowing the flame to propagate freely without spatial limitations. In contrast, the constrained environment was modeled using a quartz tube, which restricted the flame's expansion and forced the reaction products to remain within a confined space. In the experiments testing open ignition characteristics, the sample was placed in a quartz crucible at the center of the sample stage and ignited from the bottom by a Ni–Cr wire powered by a direct current source (10 V). The Ni–Cr wire had a diameter of 0.12 mm and a resistance of ≈3 Ω. A schematic of the placement of the ignition wire and the sample under open conditions is shown in Figure S6 (Supporting Information). About 5 mg of thermite sample was placed in a cylindrical corundum crucible with a diameter of 3 mm. The thermite samples under open conditions were loose and uncompressed, with a packing density equal to their bulk density (Table S1, Supporting Information). The ignition process was recorded using a high‐speed camera (Photron MiniUX50). In the experiment of measuring flame propagation under open conditions, the sample was placed in a quartz container with a strip groove cavity (80 mm × 1 mm × 1 mm). One end of the sample was ignited with a nickel–chromium wire coated with ignition powder. The sample was placed in a quartz tube and the flame propagation process was recorded with a high‐speed camera to study the flame propagation characteristics under confined conditions. The ignition temperature was measured using an infrared thermal imager (ImageIR@8355, InfraTec, Germany) with a frame rate of 2000 FPS (1/4 field of view). The emission spectrum during the combustion process was measured using a spectrophotometer calibrated against a standard light source. Pressure output characteristics were measured using a sealed burst disk device (15 mL) equipped with a pressure sensor (JUFENGKEJI, JF‐YD‐205).

### Theoretical Calculations

All computational tasks in this work were conducted using the Vienna Ab initio Simulation Package (VASP)^[^ ^50^, ^51^ ^]^ employing the projector augmented‐wave method.^[^ ^52^ ^]^ The Perdew–Burke–Ernzerhof functional within the generalized gradient approximation was utilized.^[^ ^53^ ^]^ The energy cutoff was set to 500s eV. The valence electron configurations were as follows: Al 3s^2^3p^1^, Bi 6s^2^6p^3^, F 2s^2^2p^5^, and O 2s^2^2p^4^.

## Conflict of Interest

The authors declare no conflict of interest.

## Supporting information



Supporting Information

## Data Availability

The data that support the findings of this study are available from the corresponding author upon reasonable request.
