# Supplementary material for: Pioneering Insights into the Reaction Kinetics of Metastable Intermolecular Composites Based on Metal Fluorides: Virtually non‐existent condensed Phase Combustion Products and Ultra‐Efficient Reactivity
Source: Adv Sci (Weinh). 2025 Feb 18;12(14):2415073. doi: 10.1002/advs.202415073 (PMC11984870; doi:10.1002/advs.202415073)
Supplement: Supplementary file 1 — Supporting Information [file ADVS-12-2415073-s001.docx]

**Supplementary material**

**Pioneering Insights into the Reaction Kinetics of Metastable Intermolecular Composites Based on Metal Fluorides: Virtually Nonexistent Condensed Phase Combustion Products and Ultra-Efficient Reactivity**

**Xuwen Liu ^1, 2^, Jingwei Li ^1, 2*^, Shenghua Feng ^3^, Yongsheng Jia ^1, 2^, Maocong Hu ^1, 4^, Yingkang Yao ^1, 2^, Jinshan Sun ^1, 2^, Quanmin Xie ^1, 2^, Hongqian Sang ^1, 4^**

1 State Key Laboratory of Precision Blasting, Jianghan University, Wuhan 430113, China

2 Hubei Key Laboratory of Blasting Engineering, Jianghan University，Wuhan 430056, China

3 School of Chemical and Blasting Engineering, Anhui University of Science and Technology, Huainan 232001, China.

4 School of Optoelectronic Materials & Technology, Jianghan University, Wuhan 430056, China

Corresponding author: Jingwei Li, Learth@jhun.edu.cn

**1. Charging parameters for flame propagation experiments under constrained conditions**

Charge density is an important factor affecting flame propagation under constrained conditions. In studying the flame propagation behavior of nano-thermite systems under constrained conditions, the percentage ($\eta_{\mathrm{TMD}}$) of the sample charge density ($\rho_{\mathrm{PD}}$) to the maximum theoretical density ($\rho_{\mathrm{TMD}}$) of the thermite system is usually used to evaluate ^[1]^, as shown in Equation (S1) shown.

|  | $\eta_{TMD}=\frac{\rho_{PD}}{\rho_{TMD}}\times100\%$ | （S1） |
| --- | --- | --- |

In the formula, $\rho_{PD}$ is the charge density (g·cm^-3^), and $\rho_{TMD}$ is the maximum theoretical density (g·cm^-3^). $\rho_{PD}$, $\rho_{TMD}$ and $\eta_{TMD}$ under the constraint conditions of each sample are shown in Table S2. $\rho_{TMD}$ is calculated from the theoretical density of each component in the system. Taking the n-Al/BiF3 system as an example, the calculation of the maximum theoretical density is shown in Equation S2.

|  | $\rho_{TMD}=\frac{1}{\frac{w_{Al}}{\rho_{Al}}+\frac{w_{{{Al}_{2}O}_{3}}}{\rho_{{\mathrm{Al}_{2}O}_{3}}}+\frac{w_{{BiF}_{3}}}{\rho_{{BiF}_{3}}}}$ | （S2） |
| --- | --- | --- |

In the formula, $\rho_{Al}$, $\rho_{{\mathrm{Al}_{2}O}_{3}}$ and $\rho_{{BiF}_{3}}$ are the theoretical densities of Al, Al_2_O_3_ and BiF_3_ respectively; $w_{Al}$、$w_{{{Al}_{2}O}_{3}}$ and $w_{{BiF}_{3}}$ are the mass fractions of Al, Al_2_O_3_ and BiF_3_ in the sample respectively, where the calculation of $w_{Al}$ and $w_{{{Al}_{2}O}_{3}}$ takes into account the active aluminum content of nano-aluminum.

Table S1. Charging conditions under the constraints of each thermite system

| Serial  number | Sample  name | Bulk density  （g·cm^-3^） | $\rho_{\mathrm{PD}}$  （g·cm^-3^） | $\rho_{\mathrm{TMD}}$  （g·cm^-3^） | $\eta_{TMD}$  （%） |
| --- | --- | --- | --- | --- | --- |
| 1 | n-ABF-0.75 | 0.619 | 0.687 | 7.742 | 8.88 |
| 2 | n-ABF-1 | 0.622 | 0.681 | 7.581 | 8.98 |
| 3 | n-ABF-1.5 | 0.567 | 0.620 | 7.033 | 8.81 |
| 4 | n-ABF-2 | 0.558 | 0.589 | 6.602 | 8.92 |
| 5 | µ-ABF-0.75 | 1.399 | 1.547 | 7.742 | 20.10 |
| 6 | µ-ABF-1 | 1.432 | 1.582 | 7.581 | 20.87 |
| 7 | µ-ABF-1.5 | 1.624 | 1.890 | 7.033 | 21.33 |
| 8 | µ-ABF-2 | 1.675 | 1.849 | 6.602 | 21.97 |
| 9 | n-ABO-0.75 | 0.166 | 0.186 | 8.343 | 10.14 |
| 10 | n-ABO-1 | 0.158 | 0.171 | 8.154 | 9.52 |
| 11 | n-ABO-1.5 | 0.148 | 0.159 | 7.815 | 9.24 |
| 12 | n-ABO-2 | 0.141 | 0.151 | 7.517 | 9.14 |

[1] Weismiller M R, Malchi J Y, Lee J G, et al. Effects of fuel and oxidizer particle dimensions on the propagation of aluminum containing thermites[J]. Proceedings of the Combustion Institute, 2011, 33(2): 1989-1996.

**2. Chemical process of BiF_3_ synthesis**

Overall, the synthesis of BiF_3_ relies on the precipitation reaction where Bi^3+^ ions in solution react with F^-^ ions to form BiF_3_ precipitates. The production of different sizes of BiF_3_ is primarily dictated by the dissociation ability of the fluoride source used in the reaction, as outlined below:

|  | $Bi{(\mathrm{NO}_{3})}_{3}\cdot5H_{2}O\to{Bi}^{3+}+3{\mathrm{NO}_{3}}^{-}+5H_{2}O$ | (S3) |
| --- | --- | --- |
|  | ${Bi}^{3+}+3F^{-}\to\mathrm{BiF}_{3}$ | (S4) |

For the synthesis of micron-sized BiF_3_ (μ-BiF_3_), we use sodium tetrafluoroborate (NaBF_4_) as the fluoride source. NaBF_4_ has a relatively weak F^-^ ion dissociation capability, which under hydrothermal conditions, dissociates slowly:

|  | $2{Na\mathrm{BF}}_{4}+4H_{2}O\to3F^{-}+5HF+{Na}_{2}B_{2}O_{4}+3H^{+}$ | (S5) |
| --- | --- | --- |

This slow release of fluoride ions leads to a gradual nucleation process, resulting in the formation of larger BiF_3_ particles.

For the synthesis of nano-sized BiF_3_ (n-BiF_3_), ammonium fluoride (NH_4_F) is employed. NH_4_F provides a strong source of F- ions, promoting rapid nucleation:

|  | $\mathrm{NH}_{4}F\to\mathrm{NH}_{4}^{+}+F^{-}$ | (S6) |
| --- | --- | --- |

The quick availability of F^-^ ions from NH_4_F leads to a faster nucleation process, resulting in the formation of smaller BiF_3_ particles.

**3. Work function (**$\boldsymbol{\Phi}_{\mathbf{wf}}$**) and work function difference (ΔΦ)**

Table S2 $\Phi_{\mathrm{wf}}$ and ΔΦ of Al (111), Bi_2_O_3_ (201), and BiF_3_ (111).

| Surface | Φ (eV) | ∆Φ (eV) |
| --- | --- | --- |
| Al (111) | 4.02 | 0 |
| Bi_2_O_3_ (201) | 5.15 | 1.13 |
| 1f-BiF_3_ (111) | 8.02 | 4.00 |
| 2f-BiF_3_ (111) | 5.40 | 1.38 |

**4. Influence of size effect on reaction kinetics in Al/BiF_3_ system**

As demonstrated by thermal analysis, the thermite reaction occurs in the condensed phase at the interface of aluminum and bismuth fluoride. Both nano- and micro-scale thermite systems exhibit similar thermal reaction pathways, indicating a fundamental consistency in the reaction mechanisms. However, the nanoscale thermite system benefits from the small dimensions of both the oxidizer and fuel, facilitating rapid mass and heat transfer between them. This ease of interaction activates the unreacted region through thermal convection at the reaction front, thereby enhancing the overall reaction kinetics.

In contrast, within the microscale BiF_3_ system, the reaction initiates at the interface between aluminum and BiF_3_. As the combustion progresses, the substantial size difference between the BiF_3_ and Al nanoparticles—where BiF_3_ is an order of magnitude larger than the Al NPs—results in an increasing distance between the remaining BiF_3_ and Al NPs. This separation exacerbates the difficulty of mass and heat transfer between them, leading to a significantly reduced reactivity of the microscale thermite system compared to its nanoscale counterpart (Fig. S1).

The size effect, therefore, plays a crucial role in determining the reaction kinetics, with the nanoscale system exhibiting superior performance due to the enhanced interfacial reactivity and efficient heat and mass transfer.


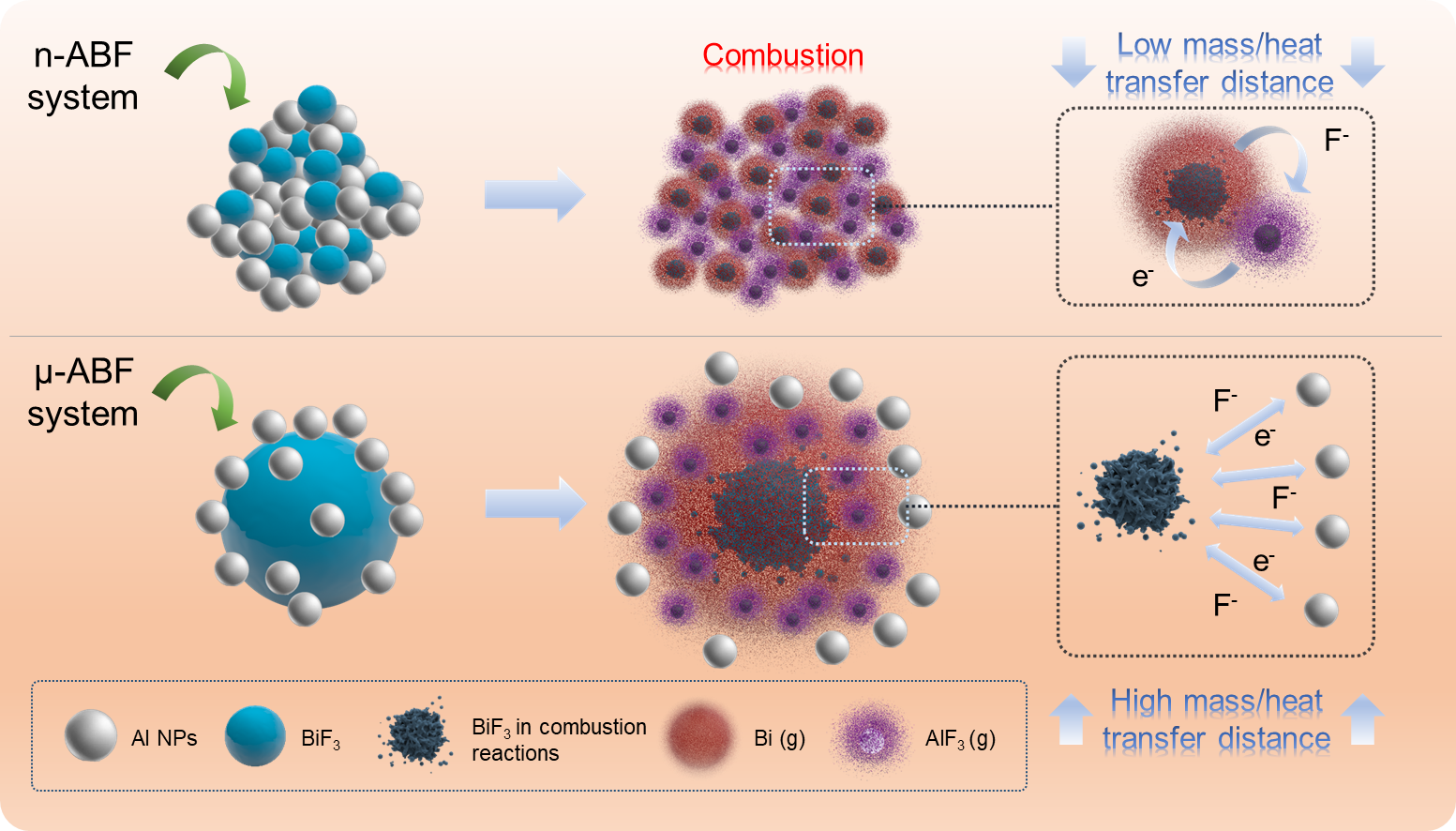


**Fig. S1.** Influence of size effect on reaction kinetics in Al/BiF_3_ system.

**5. Evolution of the reaction interface of Al/BiF_3_ and Al/Bi_2_O_3_ nano-thermite systems.**


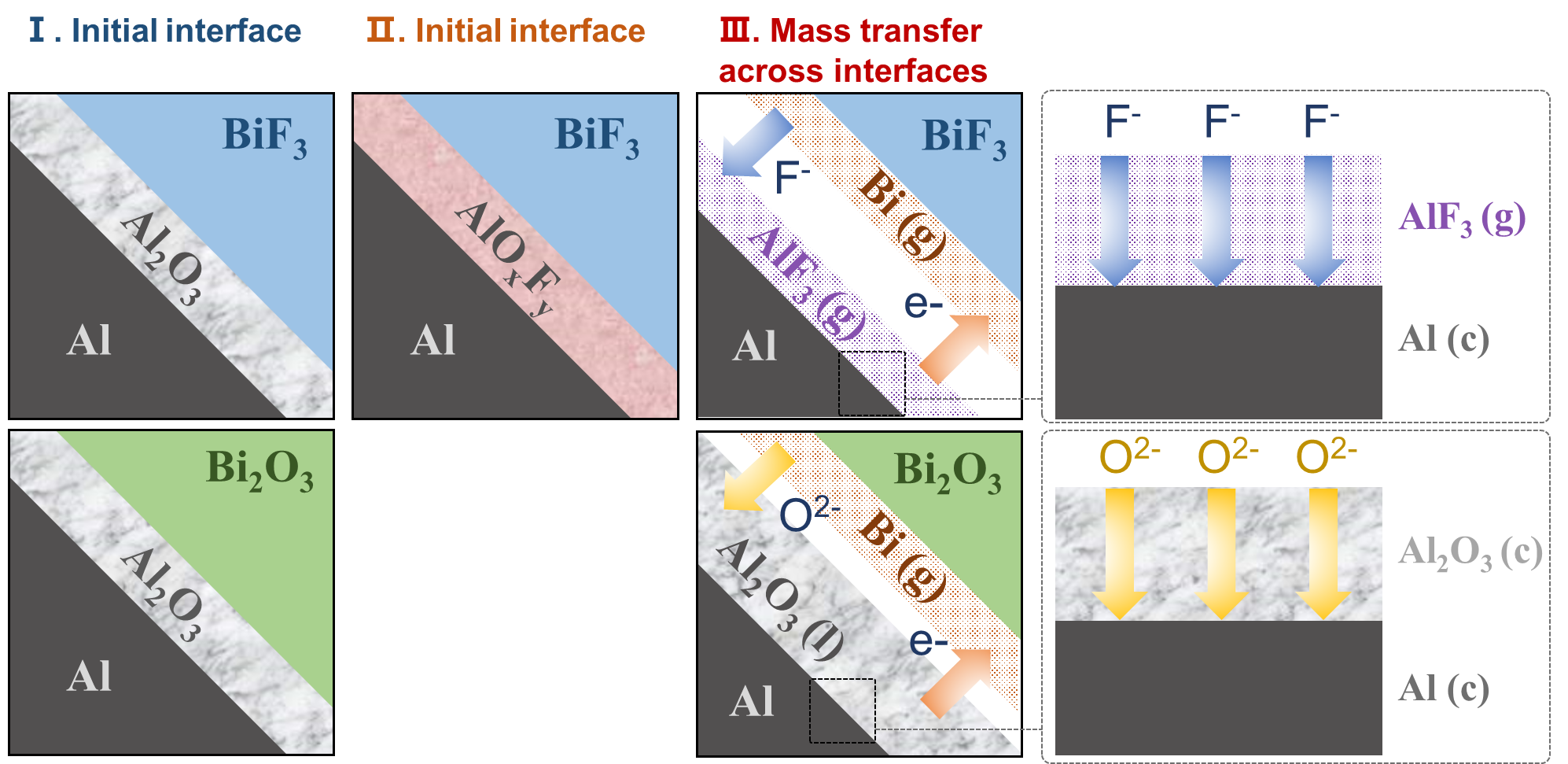


**Fig. S2.** Evolution of the reaction interface of Al/BiF_3_ and Al/Bi_2_O_3_.

**6. Comparative analysis of reaction kinetics and chemical reaction mechanisms of Al/BiF_3_ and Al/Bi_2_O_3_**.


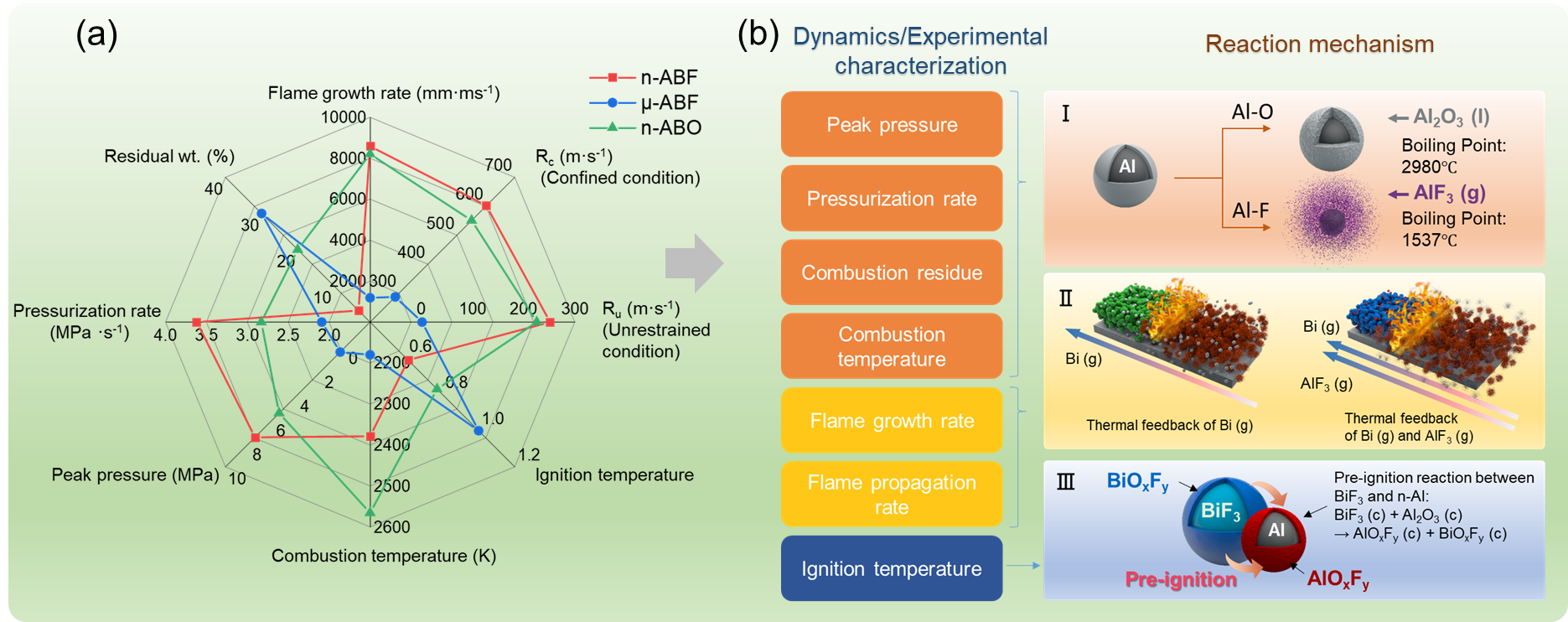


**Fig. S3.** Comparative analysis of reaction kinetics and chemical reaction mechanisms of Al/BiF_3_ and Al/Bi_2_O_3_. (a) Comparison of reaction characteristics of various thermite systems; (b) Reaction mechanisms corresponding to each reaction characteristic.

In summary, a comparative analysis of the kinetic characteristics across various nano-thermite systems reveals significant differences, as depicted in Fig. S3a. The n-ABF system, in particular, exhibits superior pressure output capabilities, including peak pressure and the rate of pressure increase. Additionally, the n-ABF system demonstrates an exceptionally low residue rate post-combustion. These enhanced performance attributes are attributed to the formation of low-boiling-point products during the energy release process in the n-ABF system. In contrast, the n-ABO system, which relies on Al-O reactions for energy release, produces Al_2_O_3_ as a combustion product with a high melting and boiling point, which is less conducive to pressure output.

The generation of low-boiling-point products in the n-ABF system not only enhances the system's pressure output performance and flame growth rate but also results in a minimal residue rate. Furthermore, the combustion temperature of the n-ABF system is slightly lower than that of the n-ABO system. This discrepancy is attributed to the lower emissivity of gaseous products compared to condensed-phase products, leading to a lower radiative temperature.

From the perspective of energy release rate, the n-ABF system displays the most potent kinetic characteristics. Whether assessing the flame growth rate under constrained or unconstrained conditions, the n-ABF system consistently shows higher values. The presence of Bi(g) and AlF_3_(g) in the combustion products of the n-ABF system contributes to the increased gas-phase products, which in turn enhance thermal convection within the reaction system and facilitate energy release.

Lastly, the fluorine-containing oxidizer in the n-ABF system endows it with a unique pre-ignition mechanism. This mechanism corrodes the Al_2_O_3_ shell prior to reaching the ignition temperature, exposing the reactive aluminum core and thereby lowering the ignition threshold of the system.

**7. Calculation of the active Al content**


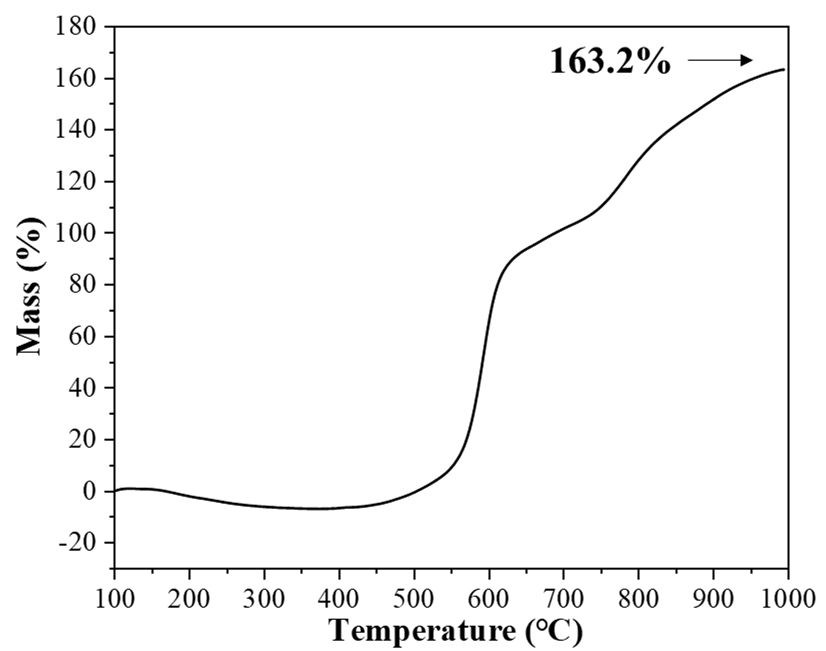


**Fig. S4.** TGA curve of n-Al in air

The active Al content was analyzed by TG at 50 °C - 950 °C, the heating rate was 10°C/min, and the air flow was 60 mL/min. The weight gain of aluminum in air is contributed by its autogenous oxidation. Therefore, the content of active Al can be calculated using formula (S7).

|  | $c\left( \% \right)=\frac{108}{96}\Delta m(\%)$ | (S7) |
| --- | --- | --- |

Where ∆𝑚 is the percent mass gain in TG. According to results of TG curve, the active aluminum content is 71.1 %.

**8. Particle size distribution of nano-Bi_2_O_3_**


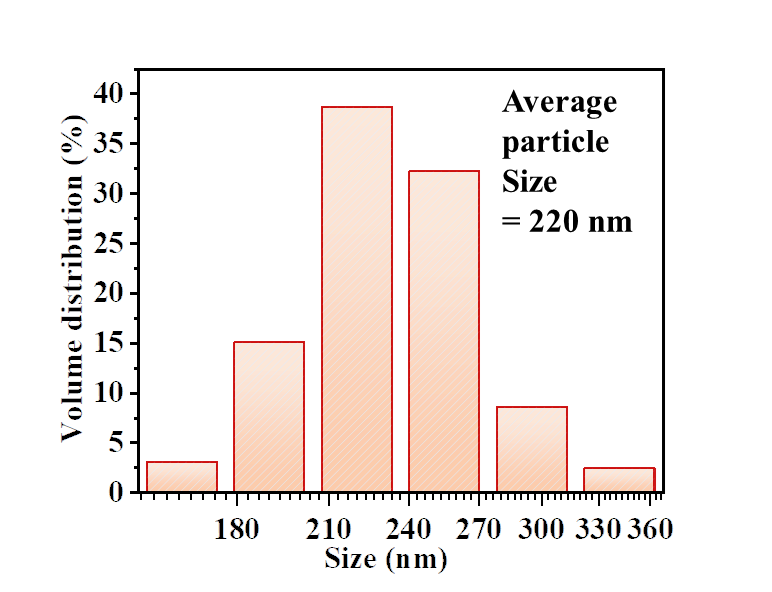


**Fig. S5.** Particle size distribution of nano-Bi_2_O_3_

**9. Evolution of the reaction interface of Al/BiF_3_ and Al/Bi_2_O_3_ nano-thermite systems.**


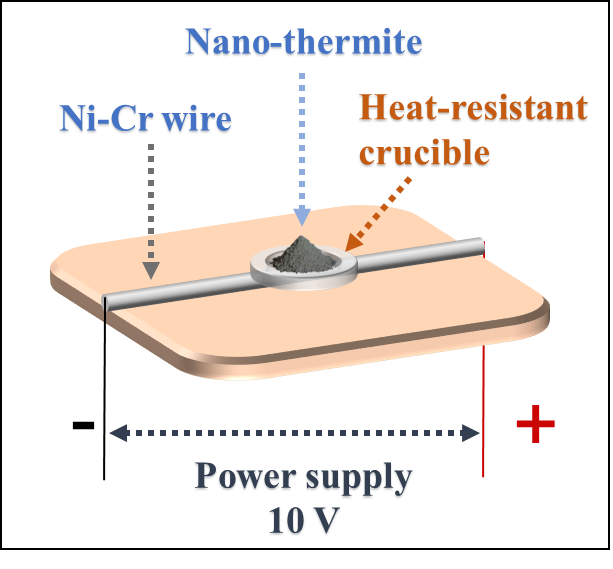


**Fig. S6**. Schematic diagram of the ignition experiment setup under open conditions

**10. Composition of n-Al/Bi_2_O_3_ nano-thermite system**

Table S3. Formula of n-ABF and µ-ABF systems.

| Serial | Sample name | | Al NPs (wt%) | n-BiF_3_ (wt%) | µ-BiF_3_ (wt%) | Φ |
| --- | --- | --- | --- | --- | --- | --- |
| 1 | n-ABF | n-ABF-0.75 | 9.3 | 90.7 | 0 | 0.75 |
| 2 |  | n-ABF-1 | 14.5 | 85.5 | 0 | 1 |
| 3 |  | n-ABF-1.5 | 25.4 | 74.6 | 0 | 1.5 |
| 4 |  | n-ABF-2 | 33.8 | 66.2 | 0 | 2 |
| 5 | µ-ABF | µ-ABF-0.75 | 9.3 | 0 | 90.7 | 0.75 |
| 6 |  | µ-ABF-1 | 14.5 | 0 | 85.5 | 1 |
| 7 |  | µ-ABF-1.5 | 25.4 | 0 | 74.6 | 1.5 |
| 8 |  | µ-ABF-2 | 33.8 | 0 | 66.2 | 2 |

Table S4. Formula of n-Al/Bi_2_O_3_ nano-thermite system

| Serial | Sample name | Al NPs *(wt%)* | n-Bi_2_O_3_ *(wt%)* | Φ |
| --- | --- | --- | --- | --- |
| 1 | n-ABO-0.75 | 11.0 | 89.0 | 0.75 |
| 2 | n-ABO-1 | 14.0 | 86.0 | 1 |
| 3 | n-ABO-1.5 | 19.7 | 80.3 | 1.5 |
| 4 | n-ABO-2 | 24.6 | 75.4 | 2 |

|  | $\Phi=\frac{{(Al/\mathrm{Bi}_{2}O_{3})}_{\mathrm{ACT}}}{{(Al/\mathrm{Bi}_{2}O_{3})}_{\mathrm{ST}}}$ | （S8） |
| --- | --- | --- |
|  | $2Al+\mathrm{Bi}_{2}O_{3}\to{Al}_{2}O_{3}+2Bi$ | （S9） |
